# Molecular mechanisms of deposit control additive inhibition by biofuels

**DOI:** 10.1039/d6cp02171a

**Published:** 2026-09-28

**Authors:** Carlos Corral-Casas, Chiara Gattinoni, Mark Brewer, Alastair Smith, Daniele Dini, James P. Ewen

**Affiliations:** a Department of Mechanical Engineering, Imperial College London South Kensington Campus London SW7 2BX UK c.corral-casas@imperial.ac.uk j.ewen@imperial.ac.uk; b Department of Physics, King's College London Strand Campus London WC2R 2LS UK; c Shell Global Solutions International B.V. Grasweg 39 1031 HW Amsterdam Netherlands; d Shell Centre York Road London SE1 7NA UK

## Abstract

Blending biofuels into gasoline can have several environmental and performance benefits. However, high ethanol concentrations are known to reduce the effectiveness of deposit control additives (DCAs) that operate by adsorption onto deposit surfaces. The fundamental physicochemical difference between fossil fuels and biofuels is that the former are mostly non-polar hydrocarbons, while the latter are predominantly oxygen-containing polar species. Here, we use molecular modelling to reveal how the presence of ethanol molecules affects the binding strength of three DCAs with markedly different chemistries to a model carbonaceous deposit from surrogate fuel blends. For a zwitterionic DCA, ethanol forms a structured solvation shell around the charged head-group, which reduces its adsorption free energy on the model deposits by around 75%. The local ethanol density at the ionic DCA head-groups saturates at low (5% volume) ethanol concentration, where most of its loss in adsorption free energy has already occurred. For a Mannich base DCA, no solvation shell forms around the DCA, even at high concentrations (85% volume). Ethanol molecules interact with the non-ionic head-group over timescales that are indistinguishable from the bulk self-network. The adsorption free energy is insensitive to ethanol concentration, even though its enthalpic and entropic components change markedly: enthalpy-driven binding opposed by entropy in iso-octane rich blends becomes weak binding with small enthalpic and entropic contributions in ethanol rich ones. Polyisobutylene succinimide DCAs are an intermediate case between the Mannich base and zwitterionic DCAs, with their adsorption free energy decreasing only at high ethanol concentrations. Our simulation results agree qualitatively with engine test results and offer a molecular-level explanation for why DCAs designed for ethanol-free gasoline are less efficient in ethanol-containing formulations. These findings provide atomistic insights to guide the rational design of DCAs for ethanol-containing fuels, which will continue to be important in the mobility landscape to enable a smoother transition towards net-zero.

## Abstract 2

Ethanol forms a solvation shell around ionic deposit control additives, limiting their adsorption on deposits in biofuels, whereas a non-ionic Mannich base is barely affected.

## Graphical abstract

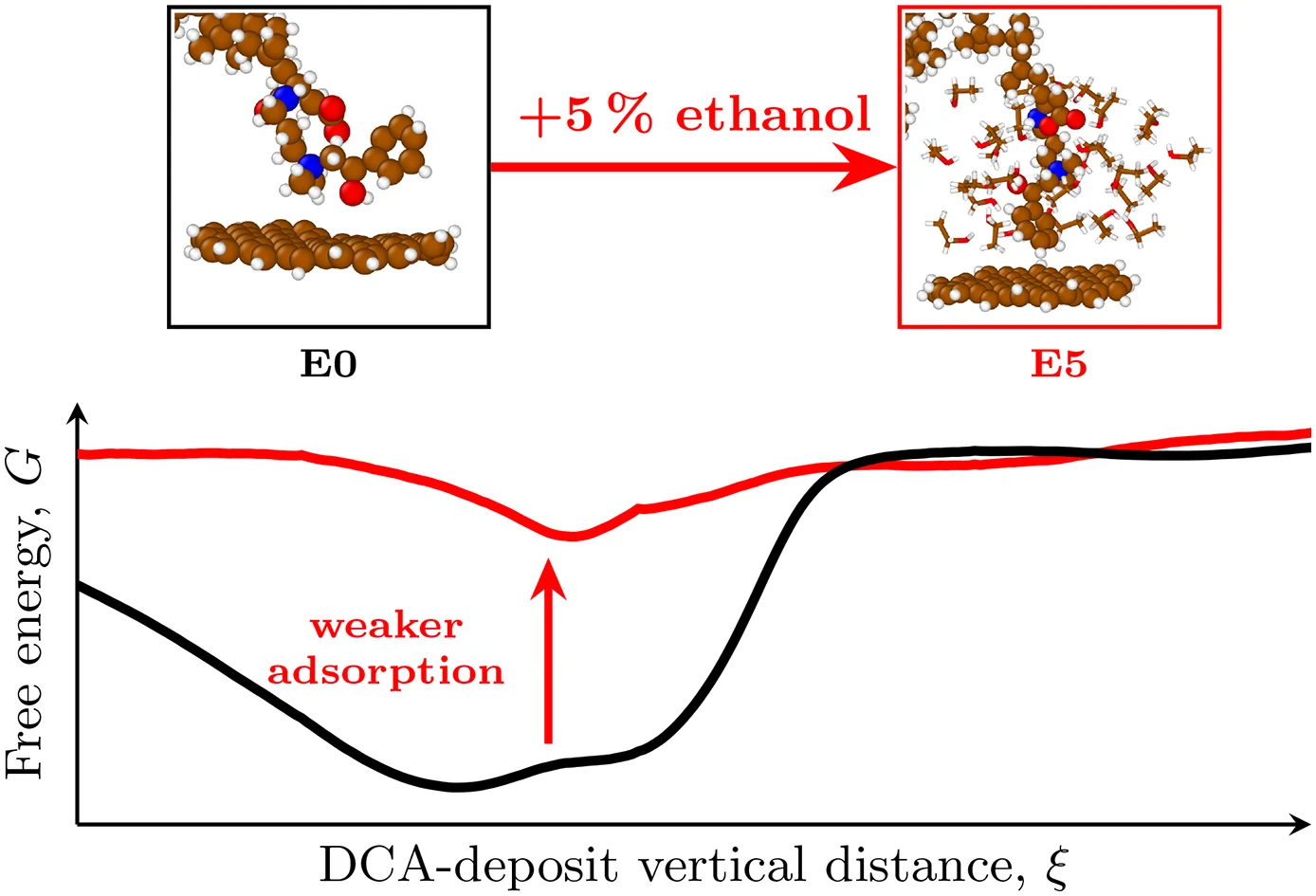

## 1 Introduction

The transportation sector remains the most dependent on fossil fuels and contributes to approximately one quarter of global CO_2_ emissions.^1^ Most vehicles are still powered by internal combustion engines that consume fossil fuels.^2^ Decarbonising this sector is crucial in the global pursuit of net-zero greenhouse gas emissions.^3^ The conversion of biomass to biofuels currently provides the most cost-effective pathway to more sustainable fossil-free liquid fuels.^4^ Depending on the feedstock and production method, biofuels can reduce life-cycle greenhouse gas emissions compared to fossil fuels,^5^ as well as improving energy independence and security.^6^ Biofuels have higher oxygen contents and polarity compared to fossil fuels,^7^ which means that they generally provide higher combustion efficiencies compared to fossil fuels.^8^ Due to more complete combustion, the use of biofuels can also reduce tailpipe emissions of some pollutants such as polycyclic aromatic hydrocarbons (PAHs).^9^

Ethanol is by far the most widely used biofuel for transportation worldwide.^10^ In most countries, ethanol is mixed with fossil fuel gasoline, with the maximum concentration depending on local government blending mandates.^11^ In most of Europe and the USA, commercial gasoline currently contains up to 10 vol% ethanol (E10).^12^ In addition to the biofuel concentration limit, fuel specifications generally also contain an oxygen limit.^12^ These concentration limits are designed to ensure that biofuel-containing blends remain compatible with existing engine technologies as well as distribution and refuelling infrastructures.^12^ Original equipment manufacturers have suggested that the majority of cars produced in Europe after 2011 are E20 tolerant,^13^ while E85 or even E100 can be used in specialist flex-fuel engines.^14^ Government biofuel blending mandates will need to increase over the coming years to reduce fossil fuel consumption.^11^ However, there are several challenges associated with increasing maximum biofuel concentrations above the current so-called ‘blend wall’.^15^

One key issue for fuel formulations is maintaining engine cleanliness, particularly the injectors.^16^ This is because formation of deposits within the fuel injector equipment can lead to a decrease in power, worse fuel economy, and increased emissions.^17^ Deposit precursors are formed from incomplete combustion during the breakdown of fuel molecules through thermo-oxidation and pyrolysis.^18^ The deposits grow through polymerisation of the degradation products followed by agglomeration until they become insoluble in the fuel.^19^ The main mitigation strategy for engine deposits is to add surfactants to the fuel formulations, known as detergents or deposit control additives (DCAs).^20^ DCAs work in two modes, preventing the agglomeration of deposit precursors to form insoluble deposits and removing those already present on metal surfaces, which are known as keep-clean and clean-up, respectively.^16^ Ultimately, the performance of these surfactants relies on their ability to (i) adhere to deposit precursors and metal surfaces *via* their polar head-group,^20^ and (ii) keep deposit precursors dispersed *via* their non-polar tail-group.^21^

The higher oxygen content and polarity of biofuels^7^ can reduce the performance of fuel additives that were designed for nonpolar hydrocarbon fuels. One challenge is that the increased polarity of the fuel can affect additive solubility.^22,23^ Moreover, for surfactant additives such as DCAs, performance may decrease due to competitive adsorption on the surface with biofuel molecules,^24^ or perhaps due to interactions of the polar biofuel species with the additives themselves. There have been many studies on the impact of ethanol concentration on the formation of injector deposits^23,25,26^ and pollutant emissions.^27–32^ Several experimental studies have also been performed on the adsorption of DCAs such as polyisobutylene succinimide (PIBSI) and its derivatives onto various surfaces from hydrocarbon solvents.^33–40^ However, the effect of biofuel concentration on DCA adsorption from hydrocarbon fuels has not been reported previously.

The development of new DCAs for fuel formulations is currently based on trial-and-error approaches, requiring many expensive, time-consuming, and polluting laboratory experiments, engine tests, and field trials.^41^ In our recent study, we showcased a novel molecular modelling approach to guide the rational design of new DCAs.^42^ We used a combination of density functional theory (DFT) calculations and molecular dynamics (MD) simulations to investigate the adsorption of DCAs onto carbonaceous deposits from hydrocarbon fuels. In that work, we were able to explain why zwitterionic surfactants outperform traditional non-ionic PIBSI additives. However, we did not consider the important role of biofuels on the adsorption process.

In this study, using the same molecular modelling approaches, we assess the adsorption of several DCA surfactant molecules on a model carbonaceous deposit from gasoline fuel blends containing different ethanol concentrations. We provide an improved picture of the fundamental mechanisms underlying the reduced DCA performance at high ethanol concentrations.^22,23^ These insights are crucial because an atomistic understanding of these phenomena could enable the rational design of advanced DCAs that are effective in high-concentration biofuel blends, which are likely to be enforced in future government mandates.^11^

## 2 Materials and methods

### 2.1 Models for biofuel systems

The methodology used here is similar to that described in our previous study.^42^ Real deposits, fossil fuels, and biofuels contain complex mixtures of molecules, which change depending on their source and processing; however, we focus on the simplest possible constituent molecules that can capture the key elements of the system. The carbonaceous deposit precursors are modelled as single PAH molecules. We selected circumovalene (C_66_H_20_), which is the largest PAH molecule identified in recent experiments that studied the molecular structure of injector deposits.^43,44^ The fossil fuel is modelled as pure iso-octane (2,2,4-trimethylpentane), which has previously been used in simulations as single-component surrogates for gasoline.^24,42^ For the biofuel component, ethanol is used since it is routinely added to gasoline. We consider a wide range of ethanol concentrations in iso-octane, from 5% (E5) to 85% (E85). These concentrations span the ranges used in current commercial fuels in most countries, as well as those that are projected to be used in the future.^12^ From previous MD simulations, we anticipate that polar ethanol molecules will self-associate into clusters in the non-polar hydrocarbon iso-octane.^45^ At high concentrations (*e.g.* E85), micro-heterogeneous mixing with segregated domains is expected.^45^

For the DCAs, we consider three different molecules, whose structures are shown in Fig. 1. The first DCA is a non-ionic PIBSI surfactant, which contains a succinimide moiety acting as a hook group, bridging the aliphatic tail-group with a tetraethylenepentamine headgroup. PIBSI has been the predominant and most effective DCA chemistry over the past few decades.^46^ The second DCA is a zwitterionic PIB surfactant with betaine chemistry,^47^ which contains both a cationic quaternary ammonium and an anionic carboxylate group. The PIBSI and zwitterionic PIB DCAs both have the same tailgroup, consisting of ten repeat units of the isobutylene monomer. The adsorption of these two DCAs on PAHs was investigated in nonpolar hydrocarbon fuels in our previous MD simulation study.^42^ The third DCA is a Mannich base surfactant, which has been shown to be more effective than other DCAs in emissions tests of fuel formulations for gasoline direct injection (GDI) engines^48–50^ and gasoline with high ethanol content.^23^ The adsorption of this Mannich base surfactant was previously investigated on iron oxide surfaces from iso-octane and ethanol/iso-octane mixtures in a recent MD study.^24^

**Fig. 1:**
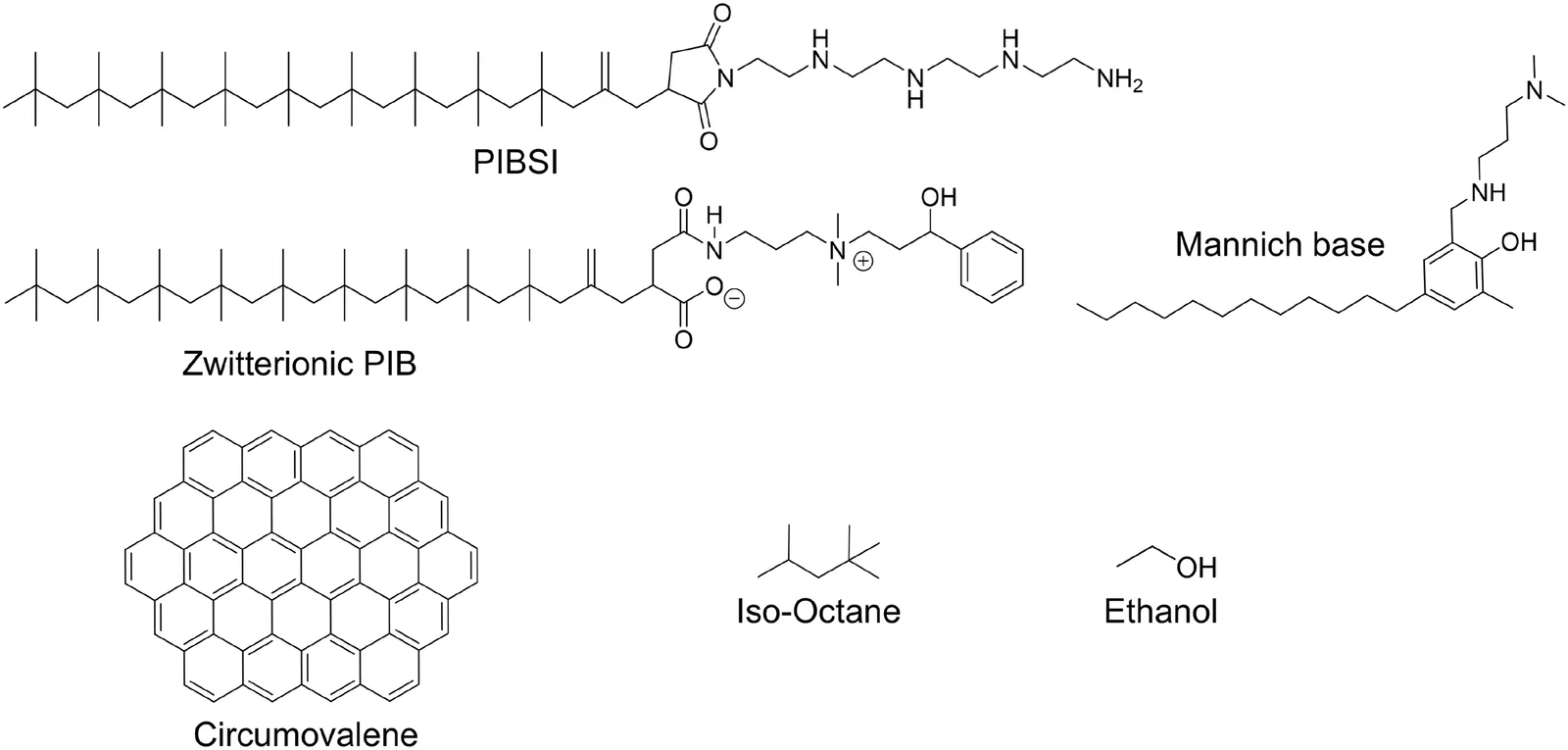
Molecular structures for the DCA (PIBSI, zwitterionic PIB, and Mannich base), fossil fuel surrogate (iso-octane), biofuel surrogate (ethanol) and PAH deposit (circumovalene) molecules considered in the MD simulations.

DCAs are known to form reverse micelles in nonpolar hydrocarbon solvents.^40,51^ In most cases, DCA adsorption can be described by the Langmuir model,^38–40^ which is indicative of monolayer adsorption. However, deviations from the Langmuir model have been observed for some DCAs at high concentrations that have been attributed to hemimicelle^33,36^ or multilayer^34,37^ adsorption. While MD simulations of micelle adsorption on solid surfaces are technically feasible,^52^ we focus on single-molecule adsorption leading to monolayer formation^53^ since these have proved to be instructive in our previous simulation study.^42^

### 2.2 Density functional theory

First-principles insights into the interactions occurring in the systems of interest are obtained using DFT. We employ DFT calculations to probe aspects of the DCA–deposit, solvent–deposit, DCA–solvent, and solvent–solvent interactions. As a result of its intrinsic cubic scaling, calculating the adsorption energy of the large, flexible DCA molecules with DFT would be computationally prohibitive.^54^ Given that the DCA tail-group is based on long alkyl chains that do not contribute significantly to the adsorption energy,^42^ we focus on the head-groups which contain the polar motifs. To fully sample the conformational space, at least ten different initial geometries are considered. These include different relative locations of the adsorbate with respect to the carbonaceous deposit, to gauge whether adsorption is favoured in the basal plane or next to the edge of the PAH sheet. DFT calculations are performed using the Vienna *ab initio* simulation package (VASP)^55–58^ with the projector augmented wave (PAW) formalism.^59^ Calculations are performed with the non-local optB86b-vdW exchange–correlation functional^60^ to account for dispersion interactions, which are important to obtain accurate adsorption energies from DFT calculations of organic molecules on various surfaces.^61,62^ A similar non-local functional has been proved to capture adsorption enthalpy values in excellent agreement with experimental data for small organic molecules binding on coronene.^63^

The plane-wave cutoff energy is set to 400 eV, with a single *k*-point in the Γ-position. The convergence criterion for the total energy in the self-consistent cycle during electronic minimisation is set to 10^−6^ eV, whereas for the forces during ionic relaxation is 10^−2^ eV Å^−1^. At least 15 Å of vacuum space is kept in all of the three Cartesian coordinates so that the plane waves die out before interacting with their periodic replicas. The adsorption energy *E*_ads_, which reflects the strength and nature of the interaction between the DCA and the PAH deposit, is computed through:

1 *E*_ads_ = *E*_sys_ − (*E*_PAH_ + *E*_gas_),

where *E*_sys_ is the total energy of the geometrically relaxed adsorbed complex, *E*_PAH_ is the total energy of the relaxed deposit, and *E*_gas_ is the total energy of the relaxed molecular fragment in vacuum.

### 2.3 Molecular dynamics

The systems for the MD simulations are constructed using the materials and processes simulations (MAPS) platform from Scienomics SARL. A single circumovalene molecule is fixed in place by constraining the positions of three of its atoms so that it cannot translate or rotate. A single DCA molecule was placed above the circumovalene at the centre of the simulation box.^42^ Afterwards, different ratios of iso-octane and ethanol molecules are randomly added using the Amorphous Builder plugin in MAPS. The number of solvent molecules added was as follows: 500 iso-octane molecules for the E0 system, 500 iso-octane and 75 ethanol molecules for E5, 470 iso-octane and 150 ethanol molecules for E10, 445 iso-octane and 230 ethanol molecules for E15, 370 iso-octane and 450 ethanol molecules for E30, and 80 iso-octane and 1260 ethanol molecules for E85. Here and throughout, *Ex* follows the fuel-blending convention: *x* volumes of ethanol per 100 volumes of blend components, measured for the pure liquids at room temperature before mixing. This fixes the ratio of molecule numbers, *N*_EtOH_/*N*_iso_= [*x*/(100 − *x*)] *V*_m,iso_/*V*_m,EtOH_, which the numbers above reproduce to within 0.5%. These numbers of molecules roughly correspond to the expected liquid density for these mixtures (∼ 0.7 g cm^−3^)^64^ for the chosen simulation box dimensions (*x*, *y*, *z* ∼ 40, 40, 90 Å). A schematic of the simulation box is shown in Fig. 2.

**Fig. 2:**
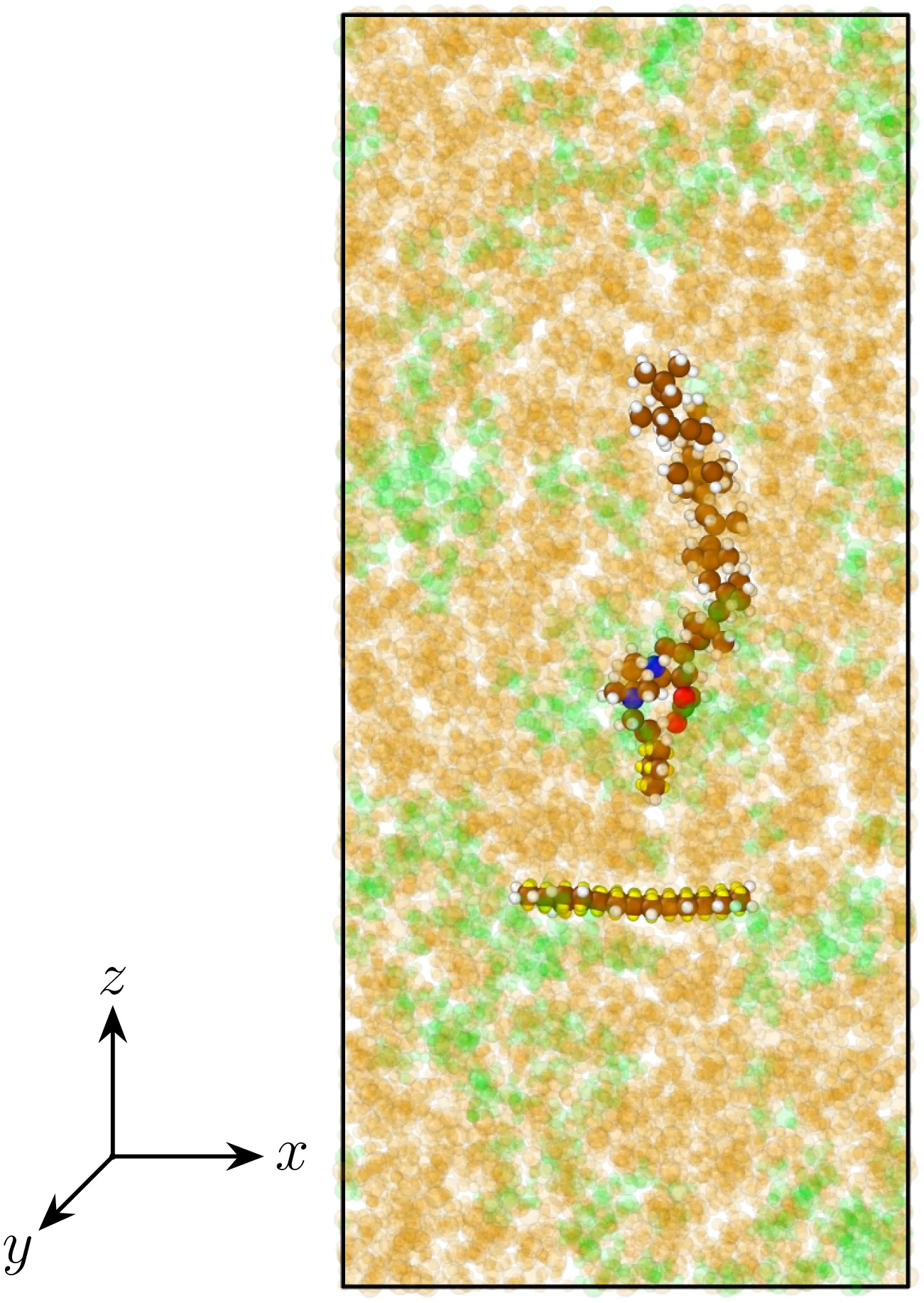
Snapshot of the system setup for the ABF-MD simulations. The deposit and the surfactant are shown using the ball-and-stick model, and the base solvent is represented with transparency, using mustard colour for iso-octane and green colour for ethanol. C atoms are shown in brown, H atoms in white, O atoms are shown in red, and N atoms in blue. Virtual π-electrons are shown in yellow. Rendered using OVITO.^65^

MD simulations are performed using the open-source Large-scale Atomic Molecular Massively Parallel Simulator (LAMMPS) software.^66^ The velocity-Verlet algorithm is used with a time step of 1 fs. Periodic boundary conditions are applied in all three Cartesian directions. The SHAKE algorithm is used to constrain the equilibrium length values for all covalent bonds involving hydrogen atoms.^67^ The non-bonded interactions include both the Lennard-Jones potential with a 12 Å cut-off and the long-range Coulomb interactions, which are solved in reciprocal space using the particle–particle particle–mesh algorithm^68^ with a relative force accuracy of 10^−5^. The systems are all modelled using the all-atom version of the optimized potential for liquid simulations atom (OPLS-AA) force field.^69–71^ The modification of OPLS-AA for Long Hydrocarbons (L-OPLS) is used for the long PIB chains.^72^ Geometric mean mixing rules, as prescribed by the OPLS force field, are used to represent the cross interactions between different atom types. We explicitly model the π-electrons with virtual particles using parameters from the INTERFACE force field.^73,74^ We showed in our previous study that this was important to accurately capture the strength of non-covalent DCA–deposit interactions for ionic DCAs, such as cation–π.^42^

The systems are first energy minimised using the conjugate gradient approach. Next, we equilibrate at 350 K and 1 atmosphere for 1 ns in the isothermal–isobaric (NPT) ensemble until the density stopped fluctuating. We choose 350 K as the target temperature since this is representative of injector temperatures during GDI engine operation^75^ and is below the boiling point of both solvents. Production runs are carried out in the canonical NVT ensemble at 350 K for at least 80 ns. A subset of simulations was also performed at 300 K and 325 K to check for temperature effects on adsorption and to decouple the free energy into its enthalpic and entropic terms.^76^ The temperature and pressure are controlled with a Nosé-Hoover^77,78^ thermostat and barostat^79^ with a time relaxation constant of 0.1 ps and 1.0 ps, respectively.

The structure of the ethanol-containing fluid around the DCA head-group is characterised through three complementary analyses, all performed using the MDAnalysis package.^80^ First, we compute the radial distribution functions (RDF) between the key functional groups in the DCA head-group and the solvent species. All RDFs presented in this work are sampled in spherical shells that are 0.2 Å wide. The RDF between a sitting particle of the head-group and atoms of type *X* in the solvent, *g*(*r*) = *ρ*_*X*_(*r*)/*ρ̄*_*X*_, is the ratio of the local number density of *X* at a distance *r* from the sitting particle to its average number density in the simulation cell, *ρ̄*_*X*_ = *N*_*X*_/*V*. To enable a direct comparison across ethanol concentrations, we plot *g*(*r*)*ρ̄*_*X*_ = *ρ*_*X*_(*r*), the local number density of *X* in units of Å^−3^.^81^ For ethanol, *X* is the hydroxyl hydrogen or the oxygen atom, and for iso-octane *X* is the methyl carbon and the density is divided by the five methyl carbons per molecule, so that it counts molecules. This intensive quantity removes the trivial scaling of the standard RDF definition with bulk concentration (*i.e.* plateauing at a value of 1) and instead shows how many ethanol molecules are found at a given distance per unit volume. Structural studies through the RDFs also allow us to derive an understanding about the size of the solvation shells, based on the integration of these profiles to produce the coordination number.^82^ Second, we quantify the number of hydrogen bonds formed in the system. In MD simulations, the geometric criteria for establishing a hydrogen bond are somewhat arbitrary because the potential energy is defined by a continuous interatomic potential. In this work, hydrogen bonds are identified when the donor–acceptor distance is less than a cut-off of 3 Å and the donor–hydrogen–acceptor angle is larger than 130°.^83^ We calculate the number of hydrogen bonds in the adsorption direction (along the *z*-axis) to understand how the hydrogen bonding changes with DCA–deposit separation. The hydrogen bonds are decomposed into ethanol–ethanol (self-network) and ethanol–DCA contributions, and normalised by the local ethanol density to yield the number of hydrogen bonds per ethanol molecule at each position. This local normalisation is necessary because the ethanol density is not uniform along the adsorption coordinate: it is enriched near the head-group and depleted near the deposit surface. The bin-by-bin normalisation isolates the per-molecule contribution, allowing a direct comparison of the hydrogen bonding character across different positions along the adsorption coordinate. Instead, dividing by the total number of ethanol molecules would preserve this spatial inhomogeneity, making it impossible to distinguish whether a region has more hydrogen bonds because more ethanol is present or because each ethanol molecule there has a higher bonding propensity. Third, the temporal persistence of the hydrogen bonds is quantified through the time autocorrelation function^84^:2
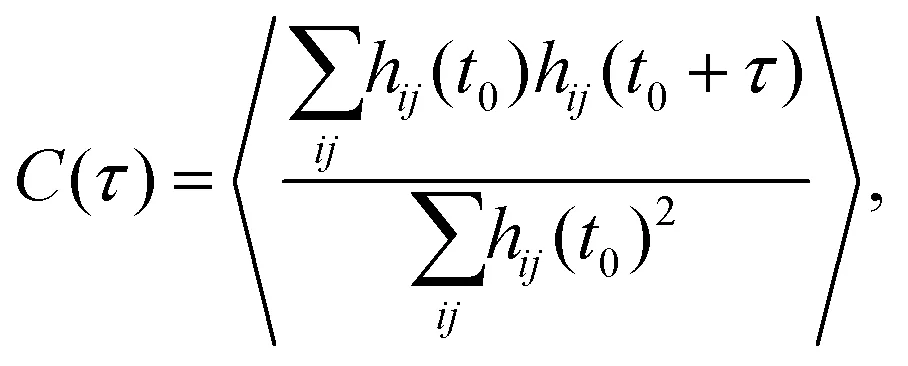
which measures the probability that a hydrogen-bonded pair present at time *t*_0_ is also bonded at a lag time *t*_0_ + *τ*. Here, *h*_*ij*_ = 1 if the pair *ij* satisfies the geometric criteria at the time origin *t*_0_, *h*_*ij*_ = 1 only if the pair has satisfied them at every sampled frame between *t*_0_ and *t*_0_ + *τ*, and *h*_*ij*_ = 0 otherwise; the angular brackets denote an average over many time origins. This is the continuous definition, in which a bond is considered broken as soon as the geometric criteria are violated and a pair that breaks and re-forms within the interval is not counted,^84^ providing a lower bound on the hydrogen bond lifetime. Pairs are identified by their hydrogen and acceptor atoms, so a hydroxyl H that switches between two carboxylate O of the same head-group is counted as a bond-breaking event. Frames are analysed every 2 ps, for lag times up to 200 ps, and *C*(*τ*) is averaged over the independent ABF strata of each system. Continuity is therefore enforced on a 2 ps grid and interruptions shorter than this are accordingly not resolved. The resulting autocorrelation function *C*(*τ*) is fitted to a biexponential decay function, *C*(*τ*) = *Ae*^(−*τ*/*τ*_1_)^ + (1 − *A*)*e*^(−*τ*/*τ*_2_)^, where *τ*_1_ captures the fast librational fluctuations and *τ*_2_ captures the slower structural relaxation of the solvation environment. Because the geometric criteria impose a sharp cut-off on a continuous potential, thermal vibrations can momentarily violate the criteria even when a pair remains physically associated, leading to an underestimation of the hydrogen bond lifetime. To verify this does not affect our conclusions, we repeated the analysis using intermittent definitions in which a bond may be absent for up to 2–30 ps without being counted as broken. The absolute lifetimes increase with the tolerance, as expected, with the ratio of DCA–ethanol to ethanol–ethanol lifetimes decreasing gradually from 6–7 to 4–5 for the zwitterionic DCA, whereas for the Mannich base it lies between 0.8 and 1.9. Since *C*(*τ*) follows a specific hydrogen-acceptor pair under a geometric criterion, it quantifies the persistence of individual hydrogen bonds; by comparing the lifetimes of the different components, we assess how much longer ethanol remains hydrogen bonded to the DCA head-group than to other ethanol molecules in the bulk self-network.

#### 2.3.1 Free energy calculations

One fundamental measure of the interactions responsible for the DCA working principle is its potential of mean force (PMF), which describes the free energy change upon adsorption as a function of the collective variable. The concept of collective variable, *ξ*, lies at the very core of enhanced sampling methodologies. For our adsorption free energy calculations, *ξ* is the *z*-projected distance between the DCA head-group and the deposit centres-of-mass. Besides accounting for the physical nature of the problem at stake, it is also important to choose the collective variable so that the biasing force is not heavily affected by stochastic noise. Therefore, *ξ* involves all non-fixed atoms in the deposit and every atom in the DCA head-group (including the H atoms affected by the SHAKE routine).^85^ The adsorption free energy reported in this work, Δ*G*_ads_, is the depth of the PMF well: the value of the free energy profile at its global minimum relative to the non-interacting plateau at large *ξ*. It quantifies the free energy of bringing the DCA head-group from the bulk solvent to its equilibrium separation from the deposit. All degrees of freedom orthogonal to *ξ*, namely the lateral (*x*, *y*) position of the DCA above the finite deposit, the orientation and internal conformation of the head-group and tail, and the solvent, are left unrestrained and are Boltzmann-averaged at each value of *ξ*. The only restraints are the harmonic walls that confine the collective variable to each window (see below) and three deposit atoms held fixed to prevent drift. The absolute value of Δ*G*_ads_ thus depends on the lateral area of the simulation cell available to the unbound DCA, but the same cell, deposit, collective variable, window scheme and reference are used for the different DCAs and ethanol concentrations, so this contribution is common to every free energy computed in this work.

The adaptive biasing force (ABF) routine^86–88^ for importance sampling is implemented using the COLVARS plugin for LAMMPS.^89^ The different parameters for promoting the algorithm convergence follow from our previous work.^42^ To further improve the free energy convergence, we stratify the relevant range of the collective variable: by confining the region of phase space that can be examined to a small range, a more comprehensive spectrum of conformations can be developed at the same computational expense. In this work, the stratification scheme is applied such that the range of *ξ* is divided into windows that are 3 Å wide, with thin bins of 0.05 Å to discretise the biasing force estimation. Quantitatively, free energies are defined with respect to the range at which they no longer change (*i.e.* the system no longer interacts directly, and the DCA has the same likelihood to remain in the bulk than to being attached to the deposit), so that the PMF is zeroed at this value. In our systems, this plateau is found at *ξ* > 12 Å and thus a minimum of five windows is considered to stratify the collective variable. Standard harmonic potentials, which act only outside the collective variable range, are applied to keep the system within the windows during the equilibration and production runs. A force constant of at least 80 kcal mol^−1^ Å^−1^ is used to avoid accumulation of samples near the borders, which would compromise the homogeneous inspection of the phase space. A comprehensive discussion on the convergence of the ABF calculations carried out to produce the free energy profiles in this work is provided in Section S1 of the SI document.

## 3 Results and discussion

### 3.1 Zwitterionic DCA

In our previous study,^42^ we showed that the zwitterionic DCA very clearly outperforms non-ionic PIBSI in terms of its binding strength onto PAH deposits from non-polar alkane solvents. In the current work, we find striking changes for biofuel surrogates characteristic of gasoline, *i.e.* iso-octane/ethanol mixtures, as shown by the adsorption PMFs in Fig. 3. The adsorption free energy for the zwitterionic surfactant from pure iso-octane, Δ*G*_ads_ = (−5.52 ± 0.25) kcal mol^−1^, already reduces considerably for E5, Δ*G*_ads_ = (−1.37 ± 0.11) kcal mol^−1^. Since DCA performance relies upon strong binding to the deposit,^42^ these observations suggest that the zwitterionic surfactant would lose much of its effectiveness in ethanol-containing fuels. A free energy obtained from MD simulations has an intrinsic resolution that is worth stating before these profiles are compared in detail. Each PMF is an ensemble average accumulated from finite sampling, so its precision is bounded by thermal fluctuations: independent replicas of the same system differ by 0.1–0.5 kcal mol^−1^ (run-to-run standard deviation), a fraction of *k*_B_*T* (∼0.7 kcal mol^−1^ at 350 K). Throughout this work, shaded bands in the PMF figures denote this run-to-run standard deviation, and quoted uncertainties are standard errors of the mean over *n* independent runs. With three independent runs per system, adsorption free energies separated by more than 0.5 kcal mol^−1^ are statistically distinguishable, whereas smaller differences, such as the precise ordering of the intermediate concentrations, fall at the resolution limit of the method and are not assigned any relevant mechanistic weight. Free energy profiles and a detailed discussion on the PIBSI results are provided in Section S2 of the SI document. Crucially, the zwitterionic DCA loses its advantage over PIBSI already at the smallest ethanol concentration in this study (E5), where the two bind comparably, and PIBSI binds significantly more strongly from E15 onwards, as we present in Table 1. In the following, we provide a comprehensive explanation for this observation: we first use DFT calculations to identify the mechanism by which ethanol suppresses the zwitterionic DCA binding, and then carry out analyses of the MD trajectories to confirm and quantify this mechanism in the liquid environment.

**Fig. 3:**
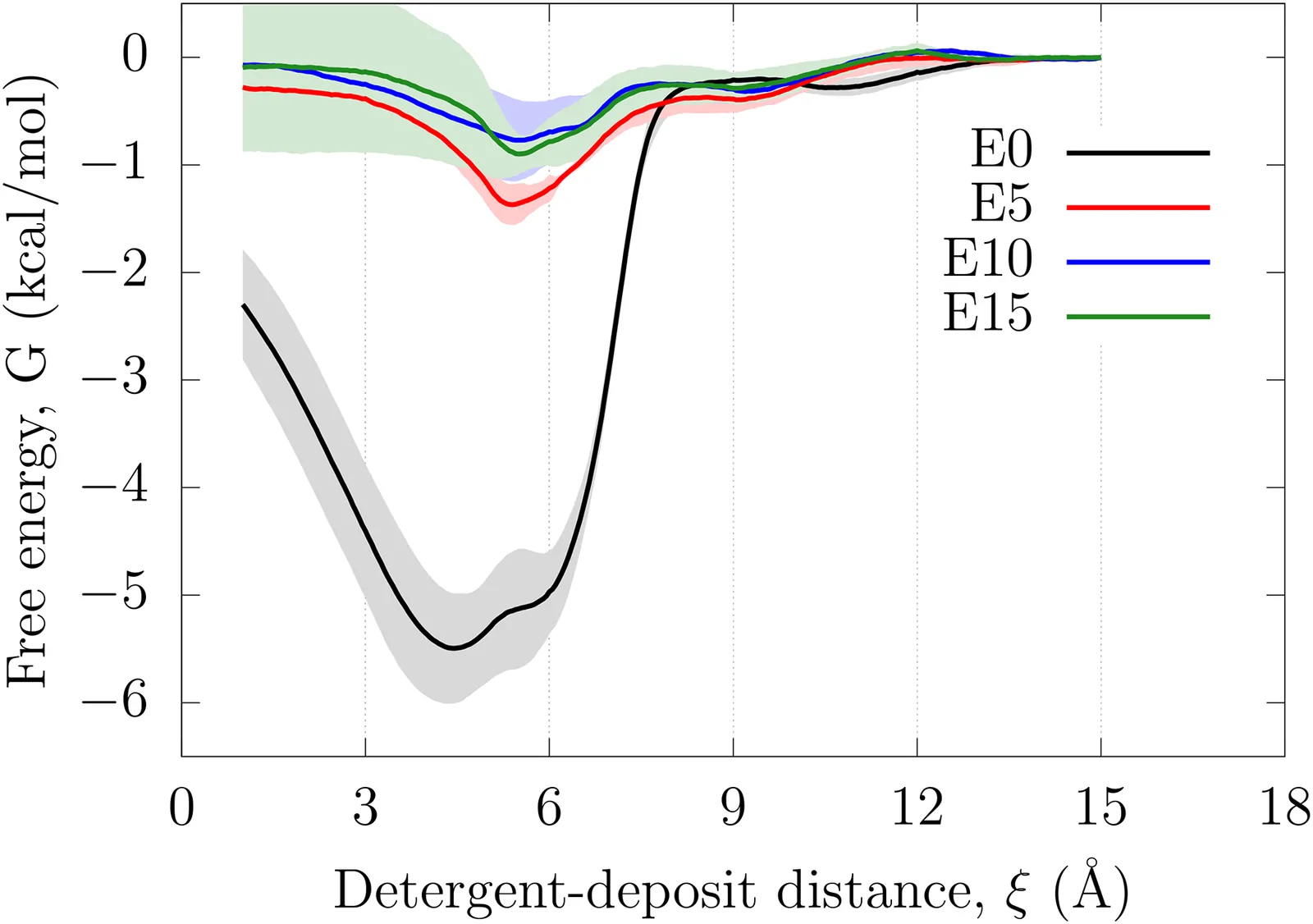
Adsorption PMF between the zwitterionic surfactant and the PAH deposit from formulations with different ethanol concentrations. Vertical dashed lines serve as a guide to the eye to point out the stratification strategy for enhanced sampling. Lines are means over independent runs and shaded bands denote ± 1 standard deviation across runs.

**Table 1:** Adsorption free energies, Δ*G*_ads_, for the three DCAs on circumovalene from different iso-octane/ethanol mixtures at 350 K, in kcal mol^−1^. Each entry is the mean over *n* statistically independent ABF-MD runs (in parentheses) of the PMF minimum, referenced to the bulk value, ± the standard error of the mean. The run-to-run standard deviation, 
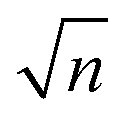
 times the standard error, is what the shaded bands in Fig. 3 and 8 show

DCA	E0	E5	E15	E85
PIBSI	−1.89 ± 0.19 (4)	−1.74 ± 0.10 (3)	−1.95 ± 0.04 (3)	−1.15 ± 0.17 (3)
Zwitterionic PIB	−5.52 ± 0.25 (4)	−1.37 ± 0.11 (3)	−0.92 ± 0.12 (3)	−0.39 ± 0.11 (3)
Mannich base	−2.38 ± 0.13 (3)	−2.22 ± 0.12 (3)	−2.06 ± 0.12 (3)	−2.14 ± 0.03 (3)

It has been known for several decades that ethanol can cause problems for detergent formulations designed for commercial fuels.^22^ For polar iron oxide surfaces, which are representative of the outer surface of steel fuel injectors, recent MD simulations have shown that polar ethanol molecules inhibit the adsorption of Mannich base DCAs by displacing non-polar iso-octane molecules and competing with the DCA for surface sites.^24^ However, for the non-polar PAH surfaces studied here, our DFT calculations on the adsorption of the solvent species on circumovalene suggest that ethanol (*E*_ads_ = −7.2 kcal mol^−1^) will not displace iso-octane (*E*_ads_ = −16.1 kcal mol^−1^). The former result is consistent with previous DFT calculations of ethanol adsorption on circumcoronene (−9.0 kcal mol^−1^).^90^ The higher adsorption strength of iso-octane than ethanol on the PAH is expected because dipole–π interactions can be weaker than dispersion-mediated CH⋯π interactions,^91^ especially considering that iso-octane is a larger molecule than ethanol. The ethanol-driven binding suppression of the zwitterionic DCA to the PAH deposit must therefore occur through a different mechanism than that observed previously for iron oxide surfaces.

We hypothesise that the polar –OH group in ethanol interacts with the ionic motifs in the zwitterionic DCA head-group, leading to the formation of a solvation shell around the DCA. This is hinted by visual inspection of the MD trajectories, where ethanol clusters preferentially aggregate around the ionic groups. To probe this mechanism from first principles, we carry out DFT calculations targeting the interactions between the zwitterionic head-group and the individual solvent species. DFT calculations show that the binding between the zwitterionic DCA and ethanol (*E*_int_ = −20.3 kcal mol^−1^) is much stronger than between the DCA and iso-octane (*E*_int_ = −8.5 kcal mol^−1^). While the absolute adsorption energy values in vacuum are dependent on the level of theory and the DFT functional, the relative energies confirm that the DCA interaction with ethanol is much stronger than that with iso-octane. The DFT calculation identifies two preferential sites in the DCA head-group for the interaction with ethanol. First, with the quaternary cation based on σ-hole interactions^92^ between the ammonium ion and the electronegative O atoms. Second, with the carboxylate anion due to the donor condition of the hydrogen in the ethanol –OH group. More details about these calculations, including the range of non-covalent interactions between the different DCA functional groups and either solvent species, are provided in Section S3 of the SI.

#### 3.1.1 Radial distribution functions

The preferential head-group association with ethanol predicted by DFT is confirmed in the liquid environment by the local number density from our MD simulations, *g*(*r*)*ρ*. The two panels in Fig. 4 reveal strikingly different solvation structures around the two aforementioned ionic motifs, reflecting the distinct nature of the underlying interactions. The COO^−^–H (ethanol) pair in Fig. 4a exhibits two well-resolved peaks: a sharp first peak at *r* ≈ 1.8 Å and a shorter second peak at *r* ≈ 3.7 Å. The narrowness of the first peak is characteristic of a directional hydrogen bond. The strong electrostatic attraction between the negatively charged DCA carboxylate group and the ethanol H in its hydroxyl group, combined with the strict angular requirements of the O^−^⋯H–O geometry, confines ethanol to a thin shell at a tightly defined distance. The distance in MD simulations is in good agreement with the equilibrium distance we observe in the DFT calculation (1.74 Å). The second peak represents a structurally different population: ethanol –OH hydrogen atoms in the second coordination shell, belonging to molecules that are hydrogen-bonded to the first-shell ethanol, rather than directly to the DCA. Because the carboxylate can only accept hydrogen bonds, this second shorter peak necessarily arises from hydrogen bond networks propagating outwards from the anion. This layered arrangement is characteristic of solvation in low-dielectric media.^93^

**Fig. 4:**
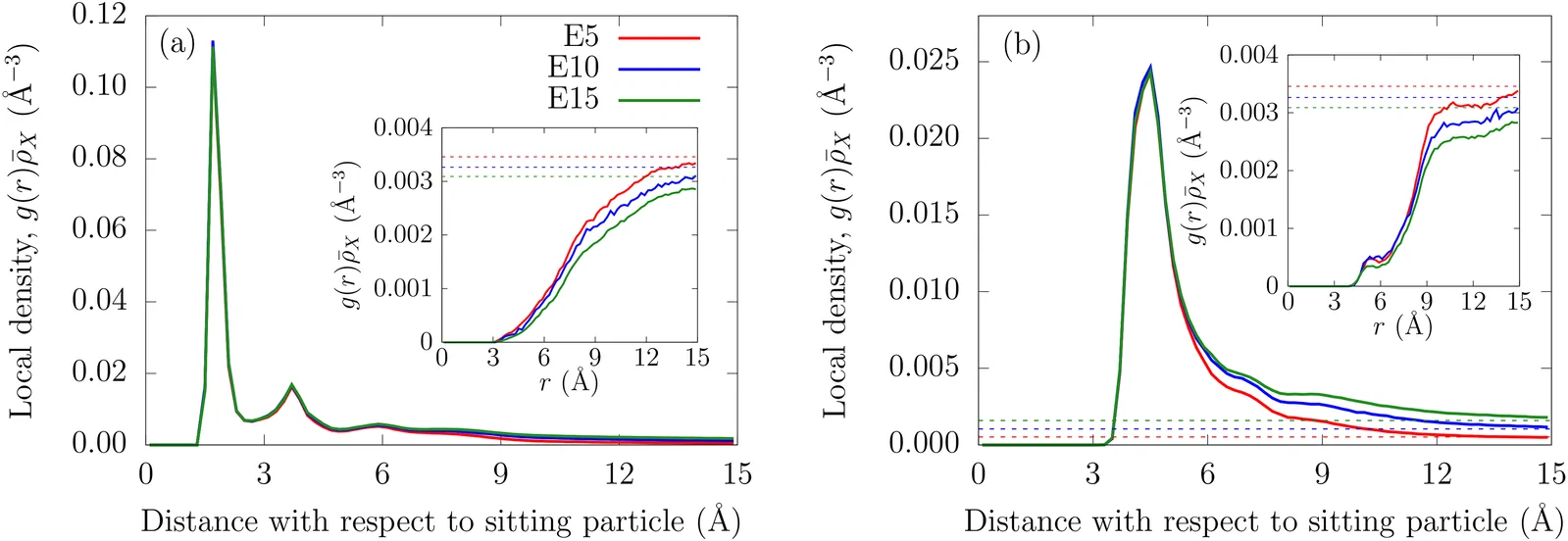
Average local number density around the zwitterionic head-group from the independent ABF-MD simulations at different windows. (a) Ethanol H – carboxylate O and (b) ethanol O – ammonium N. Horizontal dashed lines mark the average number density of ethanol molecules in the simulation cell at each concentration, counted through the hydroxyl H atoms in (a) and O atoms in (b). Insets show the same analysis for the methyl carbons of iso-octane, with dashed lines marking the average number density of iso-octane molecules in the cell.

Integrating the local number density over the first peak (the corresponding minimum at *r* < 2.5 Å) yields a coordination number of approximately two ethanol molecules per carboxylate oxygen. Extending the integration to include the second peak (*r* < 5.0 Å) gives around six ethanol molecules, with the additional atoms belonging to the secondary shell. Crucially, the local number density peak heights are nearly identical across the E5, E10, and E15 systems, even though the average number density of ethanol in the cell triples over this range. If the DCA head-group had no special affinity for ethanol, the local density would scale proportionally with the bulk ethanol availability, but instead it saturates. This indicates that the hydrogen bond acceptor sites on the carboxylate anion are already fully occupied at the lowest ethanol concentration considered (E5) and cannot accommodate more ethanol as the bulk concentration rises. Since E5 is the lowest simulated concentration, the ethanol composition at which the shell is completed is not resolved by our data and may lie well below 5%: the shell holds of the order of fifteen ethanol molecules, against 75 available in the E5 simulation box.

The N^+^–O (ethanol) pair in Fig. 4b presents a qualitatively different profile: a single broad peak centred at a distance of ∼4.5 Å with no secondary shell. The contrast with Fig. 4a arises from the different nature of the intermolecular interactions. The quaternary ammonium carries no hydrogen bond donors or acceptors, and therefore its interaction with ethanol is driven by the ion–dipole attraction between the positive charge on N^+^ and the negative dipole on the ethanol O. This interaction depends on the angle between the ethanol dipole and the N^+^⋯O vector, but not on the direction from which the molecule approaches the cation: it is strongest when the oxygen end of the dipole points towards N^+^, an orientation the molecule can adopt from any position around the ion. Unlike a hydrogen bond, which fixes both the position and the orientation of the partner through the O–H⋯O geometry, the ion–dipole interactions leave the position of the ethanol around the cation unconstrained and tolerates a range of orientations, producing a broader distribution of ethanol positions and hence a wider peak. The peak is also shifted outwards because the quaternary N is sterically shielded by alkyl groups, which is consistent with the longer DFT equilibrium distance (4.7 Å). Despite the broader and less structured ethanol solvation shell, the same saturation behaviour is observed: the first-shell local density barely increases from E5 to E15. The coordination number over this broad shell is considerably larger than for the carboxylate; a difference that is primarily geometric since the spherical shell area at *r* = 4.5 Å is roughly six times larger than at *r* = 1.8 Å. Integrating to the region after the peak (*r* < 7 Å) gives a coordination number of 11–12 ethanol molecules around the ammonium ion, which remains constant across all studied concentrations. This is in reasonable agreement with previous MD simulations of ethanol solvation shells around quaternary ammonium ions, where the coordination number of the tetraethylammonium ion was estimated to be around 15 in pure ethanol.^82^ The larger coordination number does not imply a stronger interaction, but a less selective one, where many ethanol molecules interact from a diffuse shell as opposed to the few that form strong, geometrically precise hydrogen bonds at the carboxylate motif.

The insets in both panels of Fig. 4 complement this picture by showing the iso-octane structuring next to the ionic groups. The iso-octane local density is strongly depleted below its bulk value (dashed horizontal lines) at short distances, confirming that iso-octane is expelled from the vicinity of the DCA head-group by ethanol. The ethanol O–O and iso-octane C–C RDF pairs are provided in Section S4 of the SI, which shows the self-structure of both ethanol and iso-octane clusters. Together, these results establish that the ionic motifs of the zwitterionic head-group are preferentially solvated by ethanol. This leads to the formation of localised cybotactic regions^94^ where the ethanol molecules exhibit a more ordered arrangement than in the bulk by means of hydrogen bonds.

#### 3.1.2 Hydrogen bond analysis

To quantify the solvation mechanism and connect it to the PMF results in Fig. 3, we spatially resolve the formation of hydrogen bonds along the adsorption coordinate (*z*-direction). The ethanol–ethanol profiles shown in Fig. 5 are spatially uniform and collapse across all ABF windows within each system, confirming that the hydrogen bond network is a bulk property of the ethanol sub-phase and is not perturbed by the presence of the DCA or deposit. The per-molecule connectivity increases with concentration, from approximately 0.6 hydrogen bonds per ethanol at E5 to 0.9 at E15, reflecting that a denser ethanol sub-phase provides more hydrogen bonding partners. Nonetheless, the values remain well below pure liquid ethanol at 298 K (∼2 hydrogen bonds per molecule^95^).

**Fig. 5:**
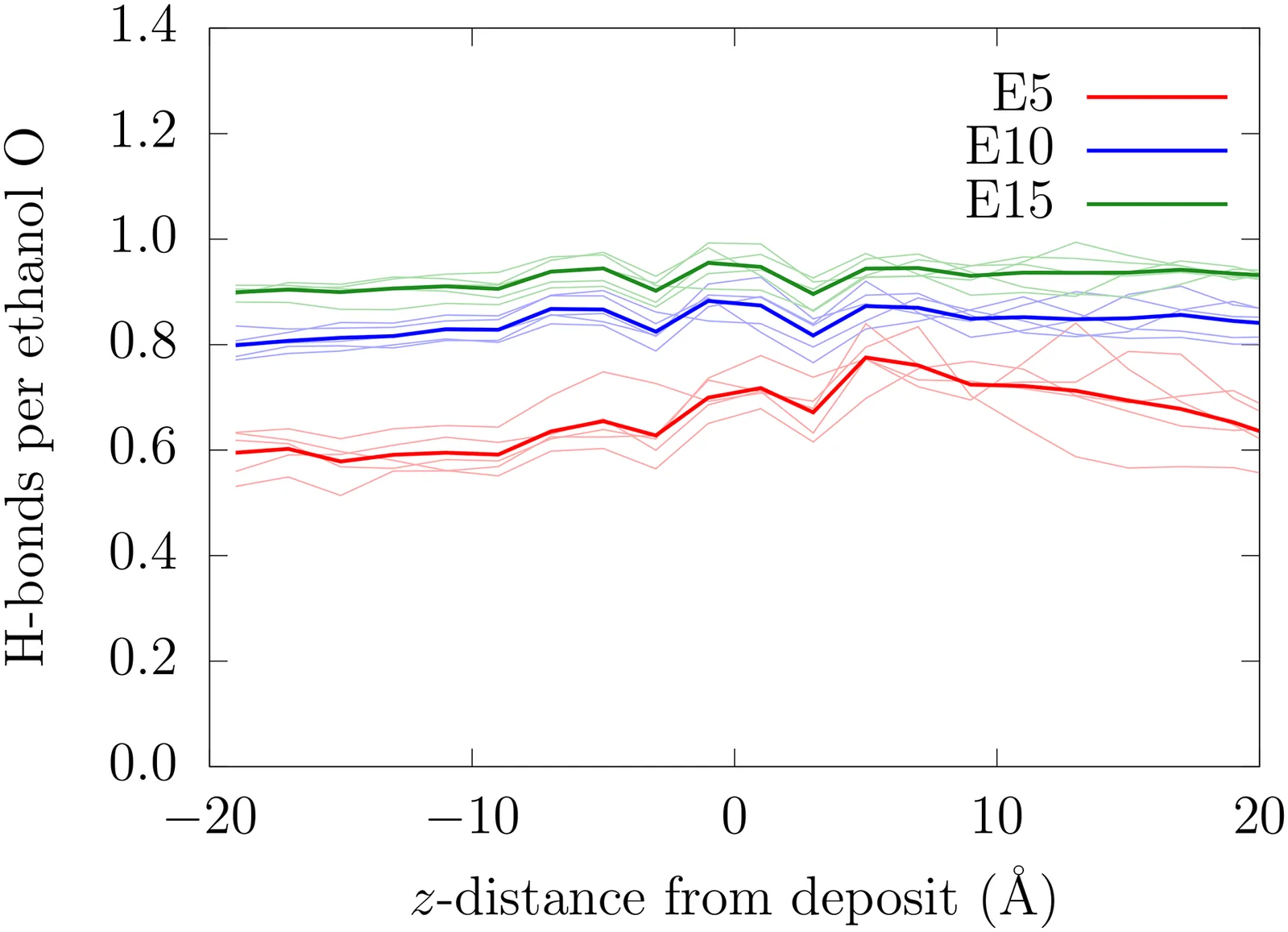
Hydrogen bonds per ethanol O atom as a function of the *z*-distance from the deposit surface. Ethanol–ethanol (self-network) contributions for E5, E10, and E15 concentrations. The solid lines are the average coming from the results at different windows, represented by the dimmed lines.

On the other hand, the ethanol-zwitterion hydrogen bonding profiles reveal a different picture, as shown in Fig. 6. Unlike the self-network, the ethanol–zwitterion hydrogen bonds are localised around the head-group. It is clear that there is preferential hydrogen bond formation close to the average DCA head-group position in the different ABF windows, which are shown by the vertical dashed lines (leftmost for the first window up to a *z*-distance of 3 Å, rightmost for the last window between 12 and 15 Å). The ethanol clusters that hydrogen bond with the zwitterionic DCA are dragged along with the head-group as it approaches the surface in the consecutive ABF windows. At low concentrations (E5), ethanol forms a higher number of hydrogen bonds per molecule (E5 ∼ 0.7) with the zwitterionic DCA head-group than at higher concentrations (E15 ∼ 0.3). This is because the solvation shell of the zwitterionic DCA is already saturated at E5, and additional ethanol molecules at higher concentrations form hydrogen bonds with the self-network (Fig. 5), rather than with the DCA. The fraction of all hydrogen bonds involving the zwitterion decreases from ∼21% at E5 to ∼6% at E15, even though the absolute number of ethanol–zwitterion hydrogen bonds increases with concentration.

**Fig. 6:**
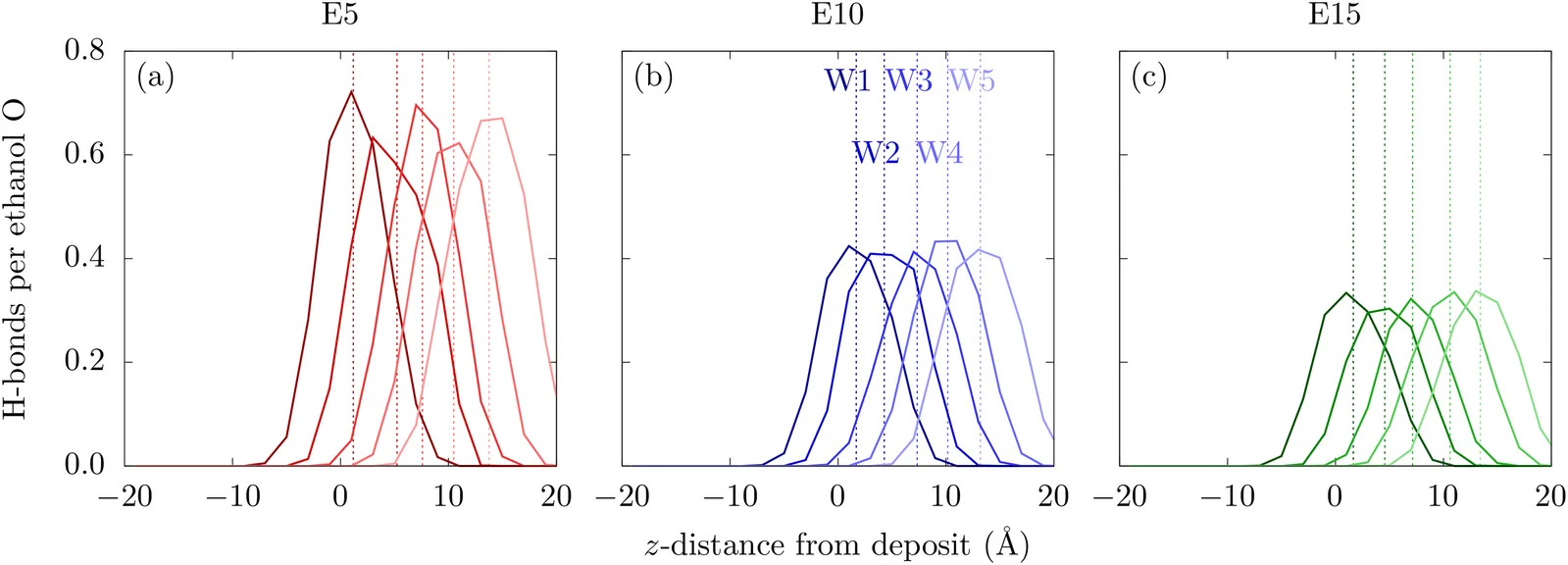
Hydrogen bonds per ethanol O atom as a function of the *z*-distance from the deposit surface. Ethanol–zwitterion (direct solvation) contributions for (a) E5, (b) E10, and (c) E15 concentrations. The vertical dashed lines represent the average location of the zwitterionic DCA head-group during the independent ABF-MD simulations within each window, W1 (0 Å ≤ *ξ* < 3 Å) to W5 (12 Å ≤ *ξ* < 15 Å).

Fig. 7 shows the continuous hydrogen bond time correlation functions, *C*(*τ*), for the two types of ethanol-driven hydrogen bond networks (with itself and with the zwitterionic DCA) presented and discussed above. For the ethanol self-network, *τ*_2_ ≈ 3.5–3.9 ps. The zwitterion–ethanol autocorrelation decays much more slowly than the ethanol self-network, yielding *τ*_2_ ≈ 23–25 ps, a six- to sevenfold difference. Thus, hydrogen bonds between ethanol and the charged head-group persist significantly longer than hydrogen bonds between ethanol molecules in the bulk, *i.e.* ethanol is retained by the ionic motifs on a timescale well beyond that of the self-network. This hydrogen bond lifetime is concentration-independent (*τ*_2_ ≈ 23–25 ps across E5 to E15), which is consistent with the solvation shell saturation established by the RDF and the spatial hydrogen bond analyses.

**Fig. 7:**
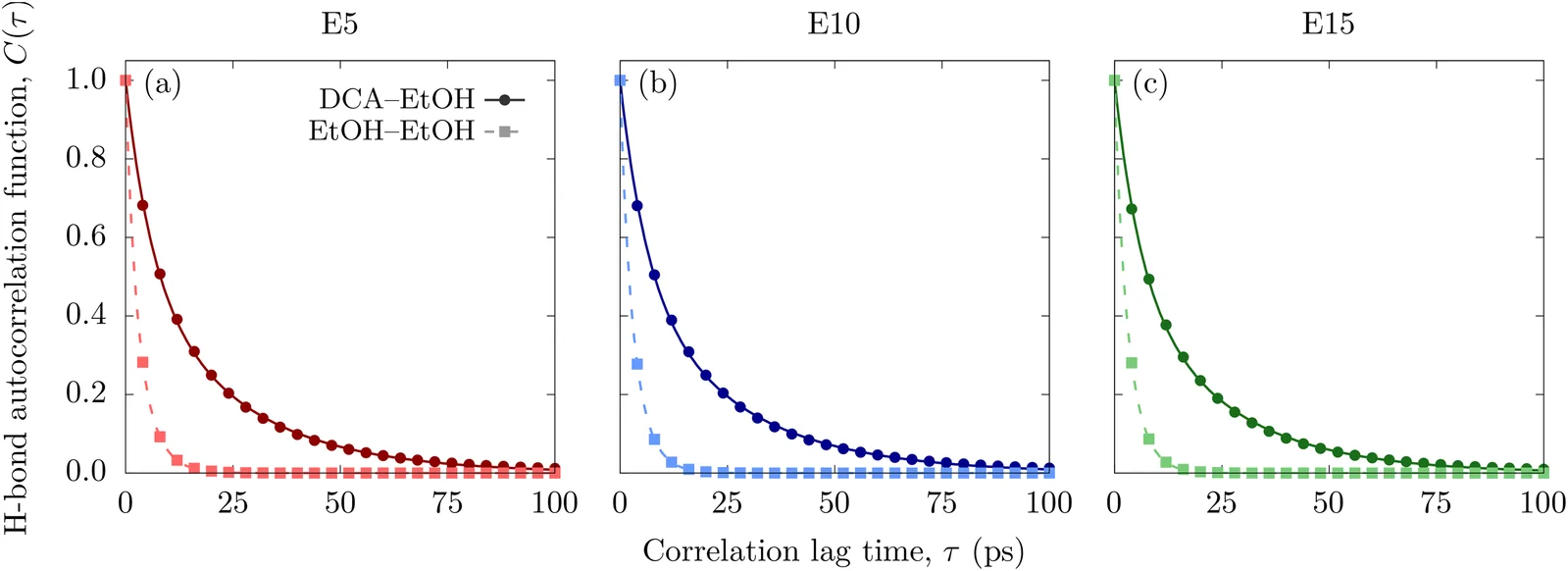
Continuous hydrogen bond time autocorrelation function *C*(*τ*) for the zwitterionic DCA at (a) E5, (b) E10, and (c) E15. In each panel, the darker circles denote the DCA–ethanol autocorrelation and the lighter squares the ethanol–ethanol (self-network) autocorrelation; solid and dashed lines are the corresponding biexponential fits. The fitted slow decay constants are *τ*_2_ ≈ 23–25 ps (DCA–ethanol) and ≈3.5–3.9 ps (ethanol–ethanol), independent of ethanol concentration.

This analysis explains the strongly non-linear PMF response in Fig. 3. The drop in adsorption free energy from E0 to E5 (from −5.5 to −1.4 kcal mol^−1^) occurs because even a small amount of ethanol preferentially clusters around the charged head-group.^93^ The solvation shell, centred on the ionic motifs, fully envelops the zwitterion and reduces its ability to bind to the PAH deposit. Adding more ethanol beyond E5 has a diminishing effect: the additional molecules mainly join the ethanol–ethanol self-network rather than further solvating the already saturated head-group. The thermodynamic preference for ethanol with respect to iso-octane is also found in our DFT simulations, with interaction energies of ethanol with the zwitterion being more than twice those with iso-octane.

### 3.2 Mannich base DCA

We have shown that the strong binding of zwitterionic DCAs to the aromatic core of the deposit is largely lost in ethanol-containing gasoline surrogates: even at E5, the zwitterionic PIB Δ*G*_ads_ = (−1.37 ± 0.11) kcal mol^−1^ no longer binds more strongly than standard PIBSI Δ*G*_ads_ = (−1.74 ± 0.10) kcal mol^−1^ on circumovalene. Alternative DCA chemistries, such as Mannich base, have been proposed as potential candidates for high-ethanol fuels.^23,24^ Its chemical structure presents some similarities with the zwitterionic one (such as aryl groups, polar heteroatoms), but without the ionic groups that become solvated by ethanol. To test whether the computational methodology deployed in this work captures the improved performance observed for this type of DCA, we perform ABF-MD simulations to derive the adsorption PMF of the Mannich base on circumovalene.

While Fig. 3 shows that the zwitterionic DCA operation is strongly affected by the presence of ethanol, Fig. 8 indicates that the adsorption of the Mannich base surfactant is insensitive to ethanol concentration at 350 K. The adsorption free energy of the Mannich base remains essentially constant in the studied range, from E0 (Δ*G*_ads_ = (−2.38 ± 0.13) kcal mol^−1^) to E5 (Δ*G*_ads_ = (−2.22 ± 0.12) kcal mol^−1^) and up to E85 (Δ*G*_ads_ = (−2.14 ± 0.03) kcal mol^−1^). It is worth noting that the absolute adsorption free energies reported for the Mannich base DCA are modest, only about 3–4× the thermal energy at the simulation temperature, while still providing a stronger adsorption on circumovalene as presented in Table 1. This reflects the simplified nature of the model deposit: circumovalene is a chemically inert PAH whose interaction with the DCA is limited to weak non-covalent forces. In reality, soot-like carbonaceous deposits are more complex entities, characterised by the presence of acidic O-containing sites arising from thermo-oxidative degradation processes during combustion. This is the subject of further studies.

**Fig. 8:**
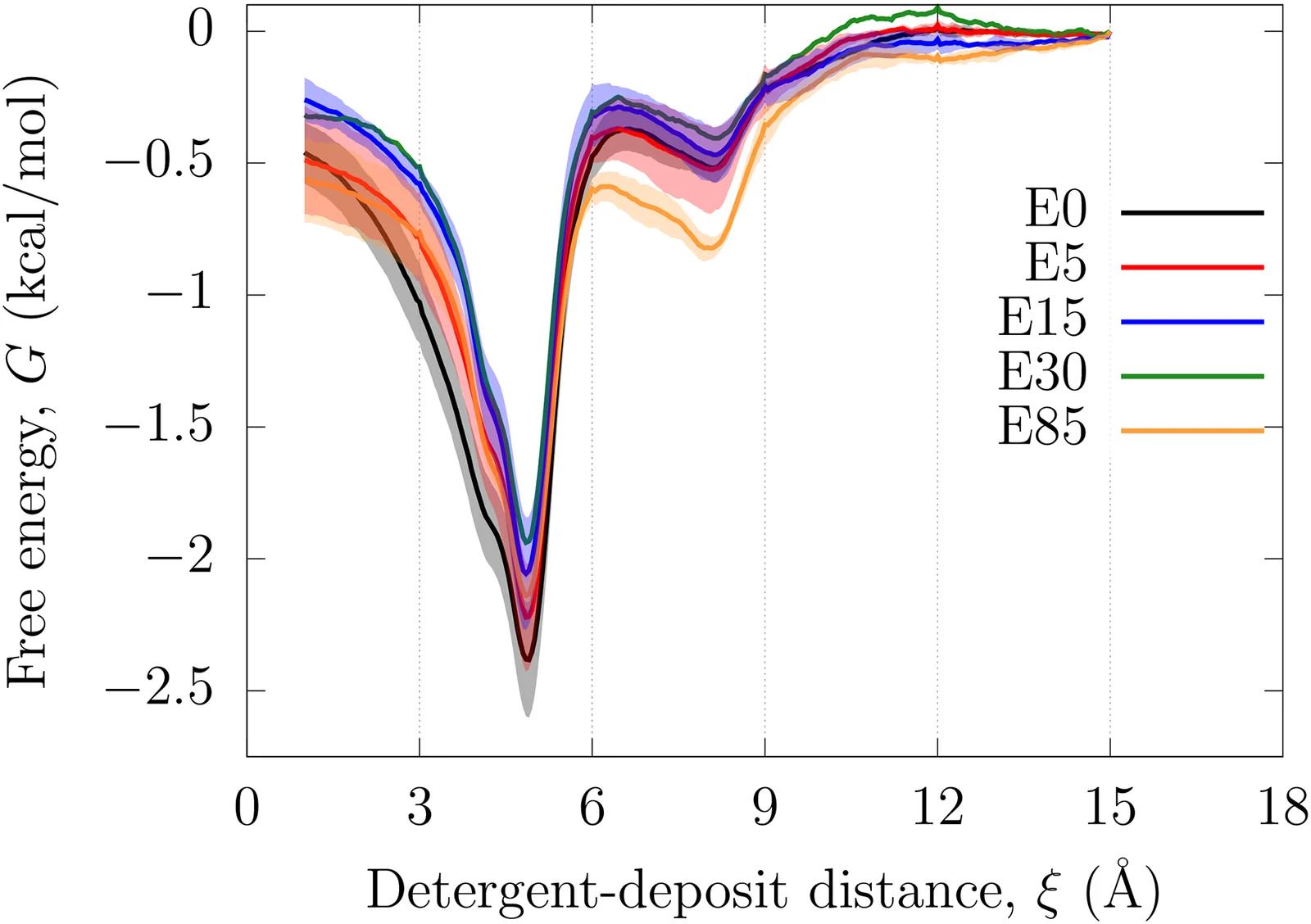
Adsorption PMF between the Mannich base surfactant and the PAH deposit from formulations with different ethanol concentrations. Lines are means over independent runs and shaded bands denote ± 1 standard deviation across runs.

Together, the PMF results suggest that, of the three chemistries, the Mannich base is the one whose binding to the aromatic core is least affected by ethanol, and hence the one expected to retain its effectiveness in high-ethanol formulations where the other two lose theirs, see Table 1. Thus, our ABF-MD results are consistent with previous engine tests that showed improved performance of Mannich bases compared to other DCAs, namely polyisobutyleneamines (similar to PIBSI) and polyetheramines.^23^ In the following, we apply the same structural analyses in the liquid region used for the zwitterionic DCA to explain the insensitivity of the Mannich base adsorption to ethanol concentration. We first use DFT to assess the headgroup–solvent interaction energies. The interaction energy between the Mannich base head-group and ethanol is half of that observed for the zwitterionic DCA (*E*_int_ = −10.2 kcal mol^−1^). Moreover, it is similar to its interaction with iso-octane (*E*_int_ = −10.1 kcal mol^−1^). This implies that there is no thermodynamic driving force for ethanol to preferentially accumulate around the Mannich base DCA and form a cybotactic region that would suppress adsorption. More details about these DFT calculations are provided in Section S5 of the SI document.

#### 3.2.1 Radial distribution functions

The absence of preferential headgroup–ethanol association predicted by DFT is confirmed by inspection of the RDFs from our MD simulations, which are presented in Fig. 9. As for the zwitterionic DCA, we plot the product of the RDF with the average number density of ethanol in the cell to enable a direct comparison across different ethanol concentrations. We chose two functional groups representing different spatial locations of the Mannich DCA: the phenolic O in the hydroxyl group attached to the aromatic ring and the N in the terminal tertiary amine. The ethanol O–O RDF is not shown here since it was found to have an identical behaviour to the zwitterionic case, which confirms that the ethanol self-network is a bulk property independent of the DCA chemistry.

**Fig. 9:**
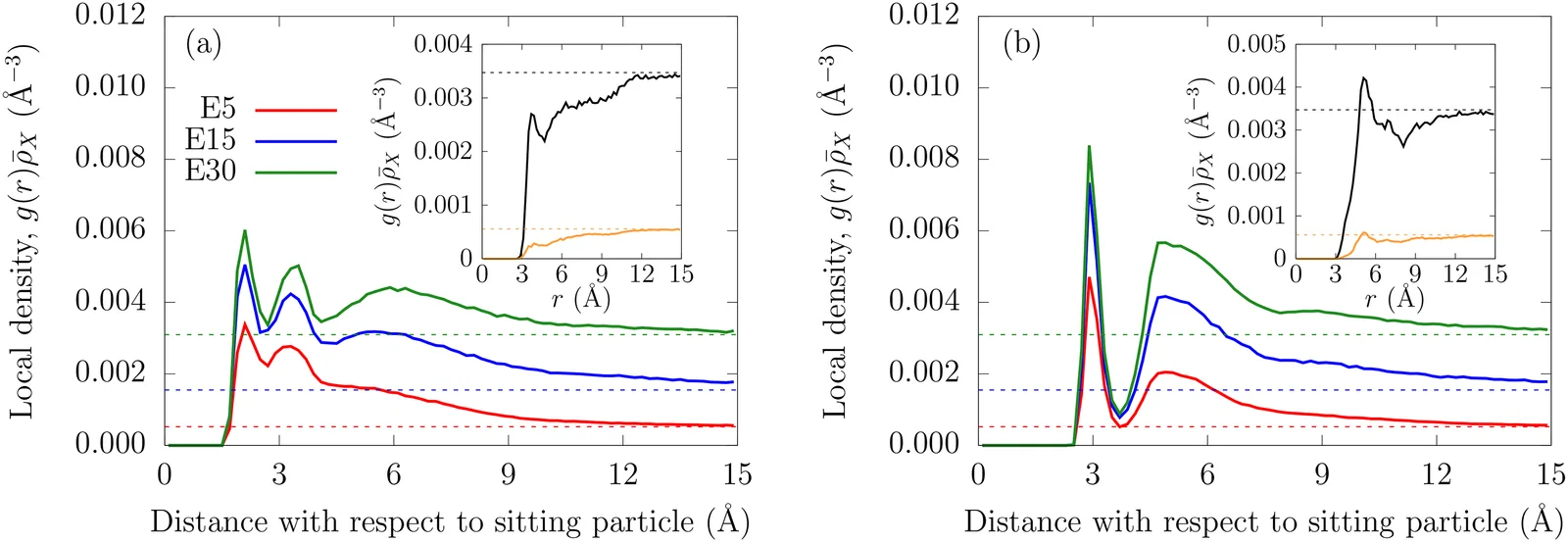
Average local number density around the Mannich head-group from the independent ABF-MD simulations at different windows. (a) Ethanol H – phenolic O and (b) ethanol O – amine N. Horizontal dashed lines mark the average number density of ethanol molecules in the simulation cell at each concentration, counted through the hydroxyl H atoms in (a) and O atoms in (b). Insets show the same analysis for the methyl carbons of iso-octane, with dashed lines marking the average number density of iso-octane molecules in the cell.

Both panels of Fig. 9 exhibit two resolved peaks: a narrow, more prominent first peak and a broader second one, but the underlying physics differ between the two functional groups stated above. The phenolic O–ethanol H pair in Fig. 9a shows a first peak at ∼2.1 Å and a second at ∼3.5 Å. The first peak corresponds to ethanol acting as the hydrogen bond donor (ethanol O–H⋯O phenol) where the detected ethanol H is the bridge atom directly constrained by the hydrogen bond geometry. This produces a narrow peak at a well-defined distance, analogous to the first peak in Fig. 4a, although at a slightly larger separation because of the weaker potential of a neutral hydrogen bond compared to the ionic one at the carboxylate. Integration of the first peak (*r* < 3 Å) yields a coordination number of only 0.24 H atoms at E5, increasing to 0.38 at E30; in contrast to the betaine carboxylate, where the same quantity is 2.0 and concentration-independent. The second, broader peak arises from the amphifunctional nature of the phenol OH group, which can also act as a hydrogen bond donor (phenol O–H⋯O ethanol). In this configuration, the hydrogen bond constrains the phenolic H and the ethanol O, but the ethanol H that we are probing sits at a distance that depends on the ethanol rotational state. This orientational freedom broadens the peak with respect to the first one and shifts it to a larger distance. That both peaks arise from the same first-shell interaction is supported by the observation that their intensity ratio remains constant (*g*(*r*)*ρ*) across concentrations.

The amine N-ethanol O pair in Fig. 9b, computed for the terminal tertiary amine, shows a first sharper peak at 2.9 Å, consistent with the DFT result (2.83 Å), and a broader second peak centred at ∼4.9 Å. The first peak corresponds to the direct hydrogen bond where ethanol donates to the amine lone pair. Its narrow shape reflects the geometric constraints of the O–H⋯N hydrogen bond, analogous to the acceptor-mode peak in panel (a). Since the tertiary amine bears no hydrogen, it can only work as an acceptor and there is no alternative bonding mode that could produce a second peak at a different distance. The coordination number within the first shell (*r* < 4 Å) is 0.32 at E5 and 0.56 at E30, increasing proportionally with the bulk ethanol rather than saturating. The broader feature at ∼4.9 Å could, in principle, arise from two sources: a genuine second coordination shell of ethanol, propagating outward from the amine, or ethanol in the first shell of the nearby secondary amine. The intramolecular N–N separation within the Mannich DCA coincidentally matches the second peak position (∼4.9 Å). To distinguish between these, we decomposed the ethanol population by proximity to the secondary amine: excluding all ethanol O atoms within the first shell of the secondary nitrogen removes less than 10% of the second peak intensity associated with the tertiary amine. This confirms that the broader peak predominantly represents a genuine second coordination shell around the tertiary amine. This interpretation is further supported by the concentration scaling: the second-to-first peak ratio grows from E5 to E30, as the increased availability of ethanol disproportionately populates the second shell relative to the first.

Despite the clear short-range structure in both panels, a key feature distinguishes the Mannich RDFs from the zwitterionic ones in Fig. 4: the local number density magnitudes scale linearly with the average ethanol number density. Ethanol molecules arrive at the Mannich head-group in proportion to its availability, with no anomalous enrichment. This is in stark contrast with Fig. 4, where the peak magnitudes collapse despite the ethanol number density tripling from E5 to E15. The linear scaling for the Mannich case can be traced to the comparable DFT interaction energies: the Mannich–ethanol interaction is of similar magnitude to the Mannich–iso-octane case, so there is no thermodynamic driving force for selective ethanol accumulation. In the zwitterionic case, the twofold excess of the ethanol interaction over iso-octane is what creates the saturated cybotactic shell that suppresses adsorption.

The insets of Fig. 9 show the iso-octane structuring next to the Mannich functional groups for two limiting cases: E0 (pure hydrocarbon, black) and E85 (mostly ethanol, orange). At E0, the iso-octane local density rises steeply near the head-group, approaching its bulk value (dashed line) by ∼ 10 Å. At E85, the iso-octane density is much lower throughout, reflecting its dilution by ethanol. Notably, the iso-octane is not preferentially expelled from the head-group surroundings; its local density simply tracks the bulk, consistent with the absence of a cybotactic region. This contrasts with the zwitterionic case, where the iso-octane insets show depletion well below the bulk value at short distances, confirming that ethanol actively displaces iso-octane from around the charged head-group. Intramolecular interactions between the –OH group in the aromatic ring and the secondary amine in the beginning of the headgroup (*e.g.* both motifs are hydrogen bond donors and acceptors) limit the space available for the solvent, particularly near the aromatic ring. Ethanol, owing to its smaller size, can be squeezed into this region more efficiently than the bulkier iso-octane molecule. Steric effects are not as prevalent in the terminal N amine, and that is why the iso-octane local density shows an oscillatory behaviour.

#### 3.2.2 Hydrogen bond analysis

To directly test whether the Mannich head-group creates a cybotactic region,^94^ we apply the same spatially resolved hydrogen bond analysis used for the zwitterionic DCA. Hydrogen bonds are decomposed into ethanol–ethanol (self-network) and ethanol–Mannich (direct solvation) contributions, and normalised by the local ethanol density at each *z*-position. As for the zwitterionic case, this removes the contribution of the spatially varying ethanol concentration and isolates the per-molecule hydrogen bonding propensity, enabling a quantitative comparison between the two DCA chemistries. The normalised ethanol–ethanol profiles shown in Fig. 10 are spatially uniform across all ABF windows and ethanol concentrations, as observed for the zwitterionic DCA. The per-molecule connectivity follows the same trend, from approximately 0.7 hydrogen bonds per ethanol O at E5 to 1.1 at E85, confirming that the ethanol network is a bulk property unperturbed by the Mannich headgroup or the finite deposit surface.

**Fig. 10:**
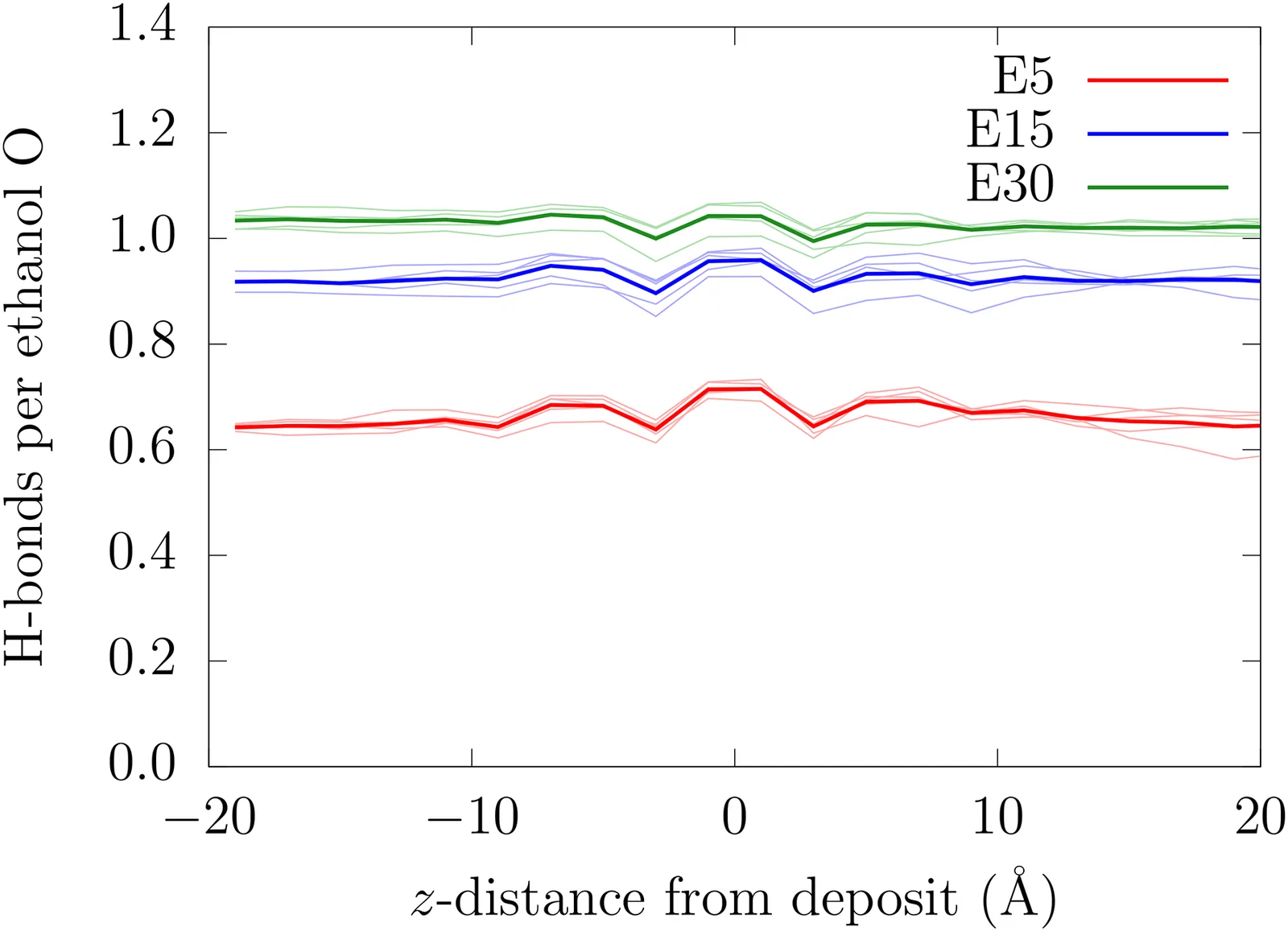
Hydrogen bonds per ethanol O atom as a function of the *z*-distance from the deposit surface. Ethanol–ethanol (self-network) contributions for E5, E15, and E30 concentrations. The solid lines are the average coming from the results at different windows, represented by the dimmed lines.

The normalised ethanol–Mannich profiles in Fig. 11, on the other hand, reveal an important difference with the zwitterionic DCA shown in Fig. 6. The peak shapes do not indicate preferential ethanol accumulation on the Mannich base head group. Rather, they reflect the short-range nature of hydrogen bonding: only ethanol molecules within a few Å of the head-group can form hydrogen bonds with it, producing a spatially localised signal even in the absence of selective enrichment. At E5, the number of hydrogen bonds to the Mannich head-group after normalisation peaks at only 0.25, compared to 0.7 for the zwitterionic DCA at the same concentration. The ethanol–Mannich fraction of total hydrogen bonds is 4.3% at E5, over five times lower than the 21% observed for the zwitterionic DCA. This ratio is remarkably constant across concentrations, with the ethanol–Mannich fraction decreasing monotonically to 0.2% at E85, which confirms a fundamental difference in hydrogen bond affinity between the two DCA chemistries. Since it is a non-ionic molecule, the Mannich base does not act as a solvation site for ethanol and the head-group only forms transient, non-specific hydrogen bonds with nearby ethanol molecules.

**Fig. 11:**
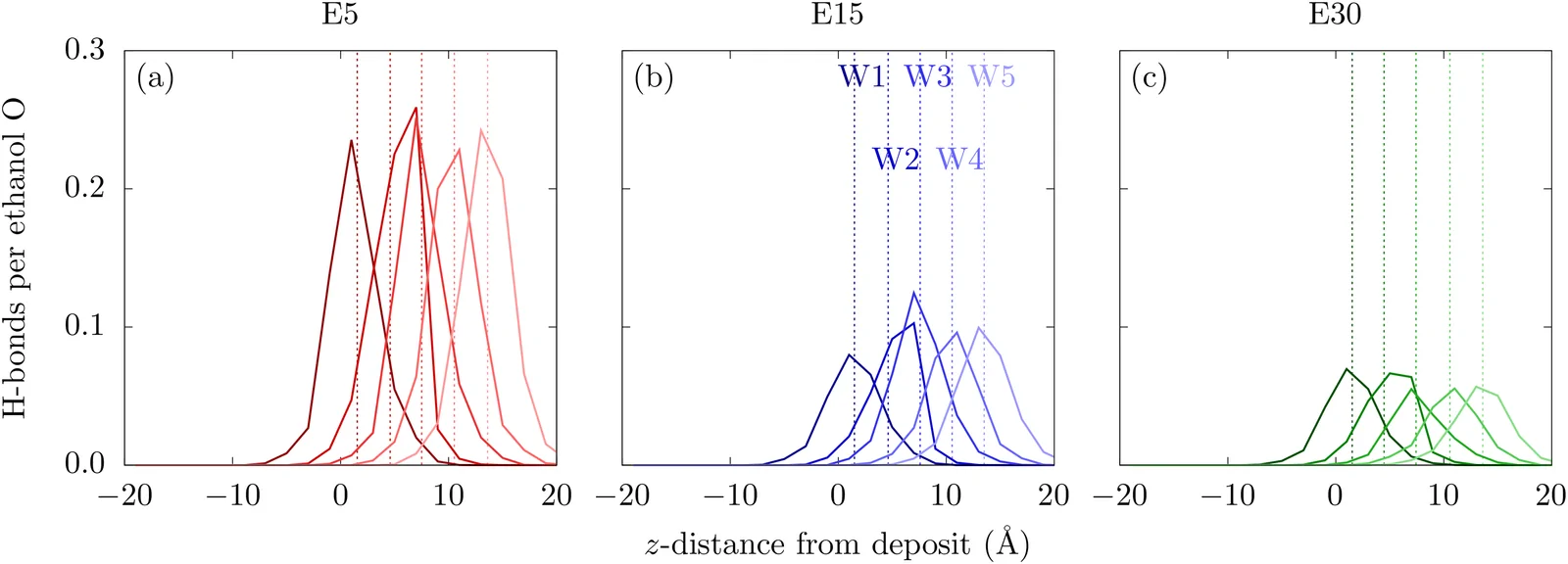
Hydrogen bonds per ethanol O atom as a function of the *z*-distance from the deposit surface. Ethanol–Mannich (direct solvation) contributions for (a) E5, (b) E15, and (c) E30 concentrations. The vertical dashed lines represent the average location of the Mannich base head-group during the independent ABF-MD simulations within each window, W1 (0 Å ≤ *ξ* < 3 Å) to W5 (12 Å ≤ *ξ* < 15 Å).

Applying the same hydrogen bond lifetime analysis (eqn (2)) to the Mannich system further demonstrates the differences to the zwitterionic DCA. Fig. 12 shows *C*(*τ*) for Mannich–ethanol (direct solvation) and ethanol–ethanol (self-network) at different concentrations. The two autocorrelation functions are virtually indistinguishable and both decay with *τ*_2_ between 2.8 ps and 3.4 ps, and the Mannich–ethanol lifetime (2.8–3.0 ps) is in fact marginally shorter than the ethanol-ethanol one (3.3–3.4 ps), giving a ratio of 0.8–0.9 that rises to at most 1.9 when intermittent definitions are used. The Mannich head-group provides no additional retention beyond what the bulk ethanol networks provide: Mannich–ethanol hydrogen bonds break on the same timescale as ethanol–ethanol ones. This result holds across all ethanol concentrations, directly confirming that the Mannich-ethanol interactions are transient and non-specific.

**Fig. 12:**
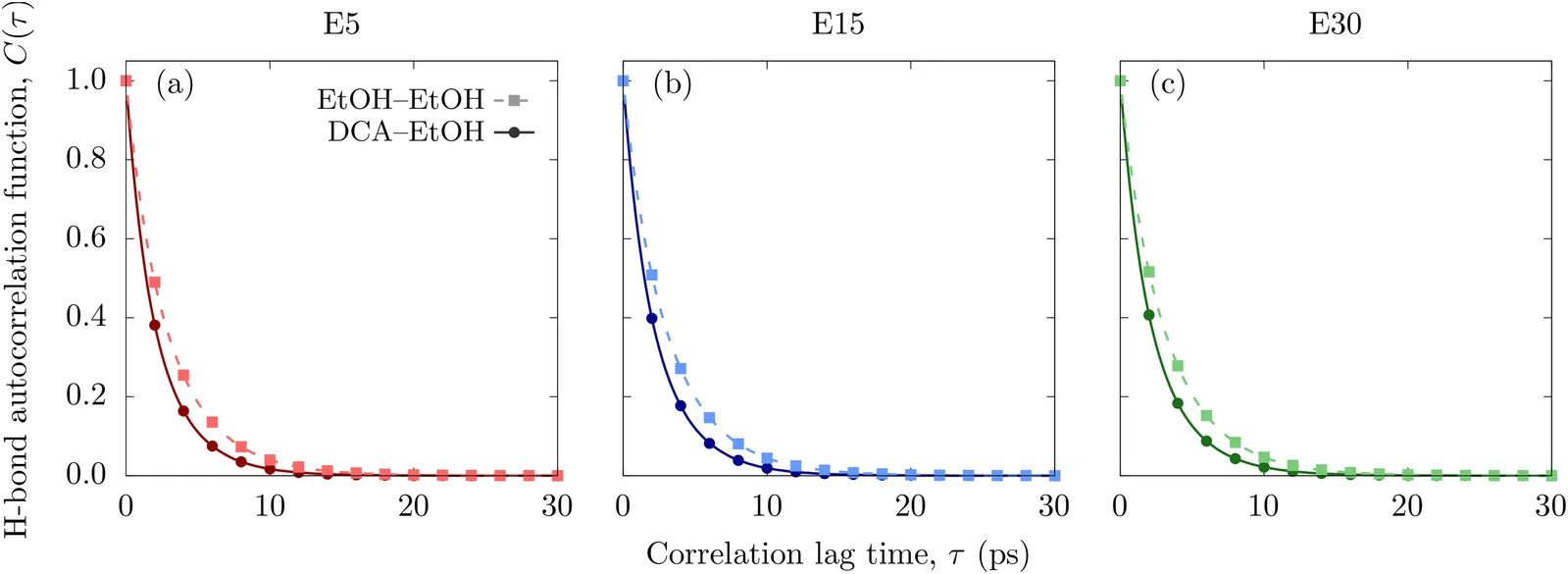
Continuous hydrogen bond time autocorrelation function *C*(*τ*) for the Mannich base DCA at (a) E5, (b) E15, and (c) E30. In each panel, the darker circles denote the DCA-ethanol autocorrelation and the lighter squares the ethanol-ethanol (self-network) autocorrelation; solid and dashed lines are the corresponding biexponential fits. Both decay quickly at all three concentrations, confirming that the Mannich head-group provides no additional retention of ethanol beyond the bulk network dynamics.

This analysis provides a mechanistic explanation for the insensitivity of the Mannich adsorption free energy to ethanol concentration. Since the Mannich headgroup does not preferentially attract ethanol, increasing its concentration does not create an increasingly unfavourable solvation penalty for removing the surfactant from bulk solution. By contrast, the zwitterionic PIB must shed its ethanol solvation shell upon adsorption, incurring a desolvation penalty that is already substantial at E5. The Mannich base experiences no such penalty because its head-group interacts with ethanol and iso-octane with essentially equal affinity, as predicted by the equivalent interaction energies calculated by DFT.

#### 3.2.3 Enthalpy–entropy decomposition of the free energy

The structural and hydrogen bond analyses above establish that the Mannich head-group does not create a cybotactic region, explaining why the adsorption free energies in Fig. 8 do not depend on increasing ethanol concentration at 350 K. However, the bulk solvent changes dramatically from E5 to E85: the ethanol hydrogen bond self-network extends (from 0.7 to 1.1 hydrogen bonds per molecule) and the dielectric environment shifts from predominantly non-polar to predominantly polar. This will inevitably affect the solubility of the DCA molecules,^23^ besides the thermodynamics of the adsorption process onto the PAH deposit. Since the solvent properties change considerably, but the free energy for adsorption remains constant, there must be a compensating mechanism. A deeper explanation on why this happens is provided in Section S6 of the SI document, while here we limit ourselves to the presentation and discussion of the key results. To investigate this behaviour, we perform an enthalpy–entropy decomposition of the adsorption free energy profiles,^76^ based on ABF-MD simulations at different temperatures:3
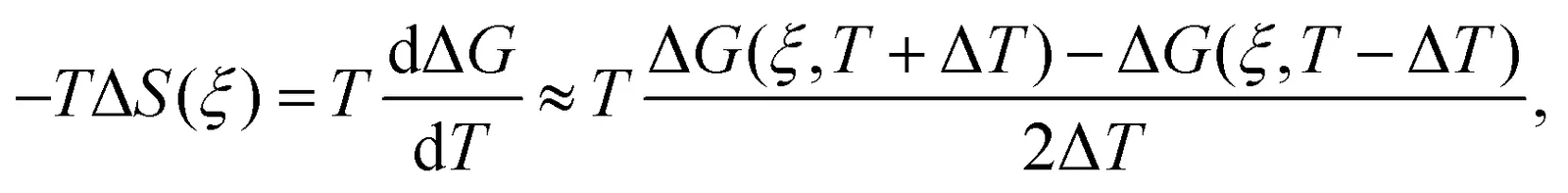
and Δ*H*(*ξ*) = Δ*G*(*ξ*, *T*) + *T*Δ*S*. Here, Δ*G*(*ξ*, *T*) = *G*(*ξ*, *T*) − *G*(*ξ*_bulk_) is the adsorption free energy profile referenced to the bulk plateau at *ξ*_bulk_ = 15 Å and the entropy, *S*, is assumed to be independent of temperature over the interval.^96^ Although strictly we compute the Helmholtz free energy in the NVT ensemble, this can be assumed equivalent to the Gibbs free energy if one neglects the pressure-volume contribution.^97^ For three equally spaced temperatures, the central difference in eqn (3) is identical to the slope of a linear fit of Δ*G*_ads_(*ξ*, *T*) = Δ*H*_ads_(*ξ*) − *T*Δ*S*_ads_(*ξ*) over the three temperatures, with Δ*H*_ads_ being its intercept. Eqn (3) is evaluated at *T* = 325 K from adsorption PMFs computed at 300, 325, and 350 K, each the mean of three independent ABF-MD runs; values quoted below are means over the three runs with their standard errors, and the statistical analysis is given in Section S6 of the SI.

The enthalpy–entropy decomposition profiles for E15 and E85 mixtures are presented in Fig. 13, which are representative of low and high ethanol concentrations, respectively. The adsorption PMFs at the three temperatures used for the decomposition are shown in the SI document (Fig. S11). The density profile for the base solvent is also included using dashed lines to illustrate the solvent layering near the deposit. Higher ethanol concentration corresponds to a stronger layering, with larger peaks and more oscillations towards the bulk of the fluid.

**Fig. 13:**
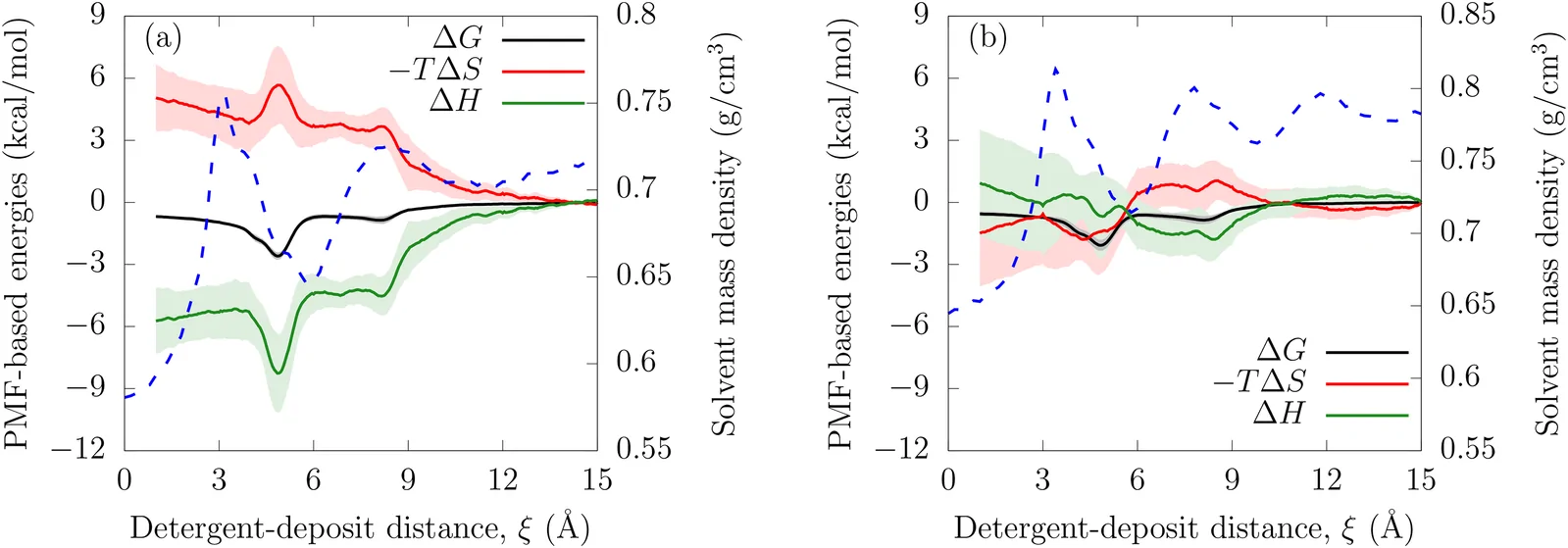
Enthalpic and entropic contributions to the adsorption PMF of the Mannich base on circumovalene from (a) E15 and (b) E85 mixtures at *T* = 325 K. Δ*G* is the mean of three independent ABF-MD runs at 325 K; −*T*Δ*S* is obtained from eqn (3) as a central difference between the 300 K and 350 K profiles, each also the mean of three independent runs, whereas Δ*H*_ads_ = Δ*G*_ads_ + *T*Δ*S*_ads_. Shaded bands are ±1 standard deviation across runs. The base solvent mass density profile is shown as a blue dashed line.

At E15, adsorption is enthalpy-driven and entropically opposed. At the adsorption minimum (*ξ* = 4.85 Å), Δ*H*_ads_ = −8.3 ± 1.0 kcal mol^−1^ and −*T*Δ*S*_ads_ = + 5.7 ± 1.0 kcal mol^−1^. Since dΔ*G*_ads_/d*T* = −Δ*S*_ads_, the change of the well depth with temperature measures the adsorption entropy directly: the well becomes shallower as the temperature rises (Fig. S11a), corresponding to Δ*S*_ads_ = −17.4 ± 3.1 cal mol^−1^ K^−1^, a loss of about 9 *k*_B_ per adsorbed molecule. A negative Δ*S*_ads_ means that adsorption weakens on heating, consistent with our previous simulations of DCA adsorption on circumovalene from pure iso-octane.^42^ At E85 the picture is qualitatively different. The well depth no longer changes with temperature within the run-to-run scatter (Fig. S11b), Δ*S*_ads_ = +4.3 ± 3.6 cal mol^−1^ K^−1^, so the entropy of adsorption is small and its sign is not resolved, and both contributions to the free energy are small, Δ*H*_ads_ = −0.7 ± 1.2 kcal mol^−1^ and −*T*Δ*S*_ads_ = −1.4 ± 1.2 kcal mol^−1^. Neither is distinguishable from zero, whereas their sum, Δ*G*_ads_ = −2.07 ± 0.15 kcal mol^−1^, is well determined. The data therefore cannot assign the binding free energy at E85 to enthalpy or to entropy; what they establish is that both |Δ*H*_ads_| and |*T*Δ*S*| are small, below 2 kcal mol^−1^, against 8 and 6 kcal mol^−1^ at E15.

What the data permits to infer is the change between the two concentrations. From E15 to E85, Δ*H*_ads_ increases by 7.6 ± 1.5 kcal mol^−1^ while −*T*Δ*S*_ads_ decreases by 7.1 ± 1.5 kcal mol^−1^. The large enthalpy–entropy compensation that characterises adsorption from the iso-octane rich solvent, a strong DCA–deposit attraction paid for by a large entropic penalty, is lost at high ethanol content: ethanol screens the enthalpic gain of the DCA–deposit contact and at the same time removes the entropic cost of binding, and the two changes nearly cancel in Δ*G*_ads_. This is why the adsorption free energy in Fig. 8 is insensitive to ethanol content, and the temperature series shows that this insensitivity is specific to the temperature of those simulations. Because Δ*G*_ads_ at E15 rises steeply with temperature while at E85 it does not, the two coincide close to 350 K (−2.06 ± 0.12 against −2.14 ± 0.03 kcal mol^−1^), whereas at 300 K the Mannich base binds more strongly from E15 than from E85 by 1.0 ± 0.2 kcal mol^−1^. As such, the enthalpy–entropy decomposition reveals a substantial change in the thermodynamics underpinning adsorption with increasing biofuel content, from enthalpy-driven binding opposed by entropy to weak binding in which neither term is large, even though the free energy itself is nearly unchanged at 350 K.

## 4 Conclusions

We have provided a molecular-level understanding of the DCA adsorption mechanism on deposit surfaces from biofuel-containing formulations. By combining DFT and MD simulations, we shed further light on the understanding of how ethanol affects the binding strength of DCAs to the aromatic core of carbonaceous deposits from gasoline surrogates. We uncovered two distinct atomic-scale mechanisms by which ethanol inhibits DCA performance, depending on the surfactant main functional groups and how these interact with the more polar species in the base solvent.

For zwitterionic PIB, the charged head-group acts as a solvation site for ethanol molecules and this cage structure inhibits the adsorption strength of the DCA on circumovalene. The solvation shell around the ionic motifs is already saturated at the lowest ethanol concentration under study (E5), and additional ethanol molecules form self-networks rather than crowding the DCA head-group. This is shown by the ethanol–zwitterion RDFs, which show two narrow peaks whose intensity does not increase at higher ethanol concentrations when being normalised by density. The hydrogen bond lifetime analysis quantifies the temporal persistence of this solvation shell: hydrogen bonds between ethanol and the charged head-group persist six to seven times longer than those within the bulk self-network, and this lifetime is concentration-independent. The twofold increase of the DFT interaction energy for the zwitterion compared to the Mannich head-group with a single ethanol molecule is amplified in the ABF-MD simulations into a fivefold difference in hydrogen bond fraction and a six- to sevenfold difference in hydrogen bond lifetime. This reflects the cooperative nature of solvation around the charged head-group, where the strong initial binding of ethanol to the ionic motifs nucleates a multi-layer hydrogen bond network that is absent for the Mannich head-group. Together, these results explain the strongly non-linear PMF response: the solvation shell is already fully formed at E5, deactivating the zwitterion and reducing Δ*G*_ads_ by 75% from E0 to E5, severely limiting its effectiveness in ethanol-containing fuels. Our results suggest that the same solvation mechanism is likely to weaken the surface adsorption not only of zwitterionic DCAs, but also of other surfactant fuel and lubricant additives containing ionic groups, such as lubricity improvers and corrosion inhibitors.^98^

For Mannich base surfactants, the picture is very different. The DFT interaction energies show that the neutral head-group shows no thermodynamic preference for ethanol over iso-octane. The ethanol–Mannich base RDFs scale linearly with bulk concentration (*i.e.* no saturation or cybotactic region) and the ethanol–Mannich hydrogen bond fraction is five times lower than for the zwitterion at the same concentration. The hydrogen bond lifetime analysis confirms that Mannich–ethanol bonds are indistinguishable from the bulk ethanol self-network, with a lifetime ratio close to unity across all studied concentrations. Without a structured solvation shell, there is no desolvation penalty upon adsorption and the PMF remains insensitive to ethanol content from E0 to E85 at 350 K. The enthalpy–entropy decomposition reveals that this insensitivity masks a substantial change in the underpinning thermodynamics: adsorption is strongly enthalpy-driven at low ethanol content, with a large compensating entropic penalty, whereas at high ethanol content both contributions collapse to values indistinguishable from zero and the modest residual binding cannot be assigned to either term within our precision. As a consequence, the insensitivity of the adsorption free energy to ethanol content holds near 350 K, while at 300 K binding from the iso-octane rich mixture is stronger by about 1 kcal mol^−1^. Thus, among the chemistries considered in this study, the Mannich base is highlighted as the one whose adsorption on the aromatic core of carbonaceous deposits is unaffected by ethanol, in agreement with recent engine tests.

These findings demonstrate that molecular modelling, combining first-principles interaction energies with MD-based free energy calculations and structural analyses in the liquid environment, can identify molecular mechanisms that underlie DCA performance and inform the design of formulations tailored to biofuel blends. These are important insights given that biofuels are expected to gain a larger market share in the short- and medium-term as governments strive for net-zero carbon emissions. Our proposed approach could contribute to reducing the reliance on expensive engine tests in the early stages of DCA development by providing an early computational screen of head-group–fuel compatibility. The inert PAH model used isolates this solvent-driven mechanism; extending the deposit model to the acidic functionalities of real deposits will allow the surface-driven contributions to DCA adsorption to be decoupled from it and the screening to be extended to the deposit chemistry. The methodology we have developed is also readily transferable to other fuel additives that rely on surface adsorption for their operation, such as corrosion inhibitors and lubricity improvers.

## Conflicts of interest

There are no conflicts of interest to declare.

## Supplementary Material

CP-OLF-D6CP02171A-s001

## Data Availability

The data supporting this article are openly available from the Imperial College London research data repository (Helix) at https://doi.org/10.82186/wnh2v-vpv11. This repository provides the adsorption PMFs underlying Fig. 3 and 8; namely, the lightweight COLVARS outputs coming from the ABF-MD simulations: the sampling counts, mean forces, and free energies. Examples of a LAMMPS input script, LAMMPS data file, and COLVARS configuration file are attached as well. The repository also provides the RDF and hydrogen bond analysis results underlying Fig. 4–7 and 9–12, alongside documented Python scripts including the main post-processing functions. One complete example MD trajectory (betaine DCA in E5 fuel) is included so that the structural analysis workflow can be reproduced from trajectory to figure on download. The full MD trajectories (tens of GB per run) and the supplementary free energy datasets (*e.g.* to produce the SI document figures) are available from the corresponding author on reasonable request. Supplementary information (SI): convergence analysis of the PMF calculations, adsorption PMF studies for PIBSI on PAH within gasoline surrogates, zwitterion-solvent DFT calculations, radial distribution studies of the solvent (iso-octane, ethanol) at different biofuel surrogates, Mannich–solvent DFT calculations, and PMFs for the Mannich base DCA at different temperatures to produce the enthalpy–entropy decomposition of the corresponding adsorption free energies. See DOI: https://doi.org/10.1039/d6cp02171a.
