# Supplementary material for: Molecular mechanisms of deposit control additive inhibition by biofuels
Source: Phys Chem Chem Phys. 2026 Sep 28. Online ahead of print. doi: 10.1039/d6cp02171a (PMC13732757; doi:10.1039/d6cp02171a)
Supplement: Supplementary file 1 [file CP-OLF-D6CP02171A-s001.pdf]

Supplementary Information (SI) for Physical Chemistry Chemical Physics.
This journal is © the Owner Societies 2026
Supplementary Information (SI) for Physical Chemistry Chemical Physics.
This journal is © the Owner Societies

Supporting Information for: Molecular Mechanisms of Deposit Control Additive Inhibition by Biofuels

Carlos Corral-Casas,*,† Chiara Gattinoni,‡ Mark Brewer,¶ Alastair Smith,§ Daniele Dini,† and James P. Ewen*,†

†*Department of Mechanical Engineering, Imperial College London, South Kensington Campus, London SW7 2BX, United Kingdom*

‡*Department of Physics, King's College London, Strand Campus, London WC2R 2LS, United Kingdom*

¶*Shell Global Solutions International B.V., Grasweg 39, 1031 HW Amsterdam, Netherlands*

§*Shell Centre, York Road, London SE1 7NA, United Kingdom*

E-mail: c.corral-casas@imperial.ac.uk; j.ewen@imperial.ac.uk

Contents

1 Convergence analysis of the PMF calculations **S2**

2 PMF studies for PIBSI within gasoline surrogates **S8**

3 Zwitterion-solvent DFT calculations **S10**

4 Structural studies of the solvent in biofuel surrogates **S12**

5 Mannich-solvent DFT calculations **S14**

6 Mannich base DCA: PMF at different temperatures S15

References S21

1 Convergence analysis of the PMF calculations

The adsorption free energies reported in this work are computed with the adaptive biasing force (ABF) method, in which the mean force along the DCA–deposit centres of mass distance ξ, the collective variable of choice, is accumulated during the simulation run and the potential of mean force (PMF) is recovered by integration, $\Delta G(\xi) = -\int \langle F_\xi \rangle_\xi \, \mathrm{d}\xi$. In enhanced sampling methodologies, regardless of the algorithm of choice, a free energy difference is only meaningful once this estimate has converged. It is worth noting that a stationary-looking profile can still conceal incomplete sampling of the $\xi-$orthogonal degrees of freedom, so we do not rely on a single criterion to judge whether convergence has been reached in our MD simulations. Instead, we verify convergence through a chain of diagnostics that follow the casual structure of the method: uniform sampling of the collective variable produces a self-averaging mean force, which is then used to integrate the potential of mean force, and statistically independent replicas reproduce it. Each diagnostic is presented side-by-side for the two detergents (betaine on the left, Mannich on the right) in Figures S1–S5, all at E15; the same checks have been applied to every PMF reported in this work.

To speed up sampling, we also partition the collective variable into adjacent 3 Å wide windows, each propagated by an independent simulation and confined to its window by harmonic walls. This is common practice, given that the time required to converge a diffusive coordinate grows as the square of its range, so that several short windows converge much faster than a single long one.[1] Each window is sampled for at least 75 ns, built up from continuation segments that we concatenate to recover the full sampling story before the windows are stitched into the complete profile over the full collective variable range. Figure S1 reports the bin-sampling histogram. The ABF algorithm supplements the force field dynamics with

a bias that opposes the mean thermodynamic force, so that at convergence the system is expected to diffuse almost freely along ξ. A consequence of this is that the system visits every bin with near-uniform frequency, with a flat histogram certifying that no region of the collective variable has been left unsampled. The observed occupancy is uniform to within a factor of about 1.5 across the entire range, with the peaks in some of the window boundaries appropriately explained below.

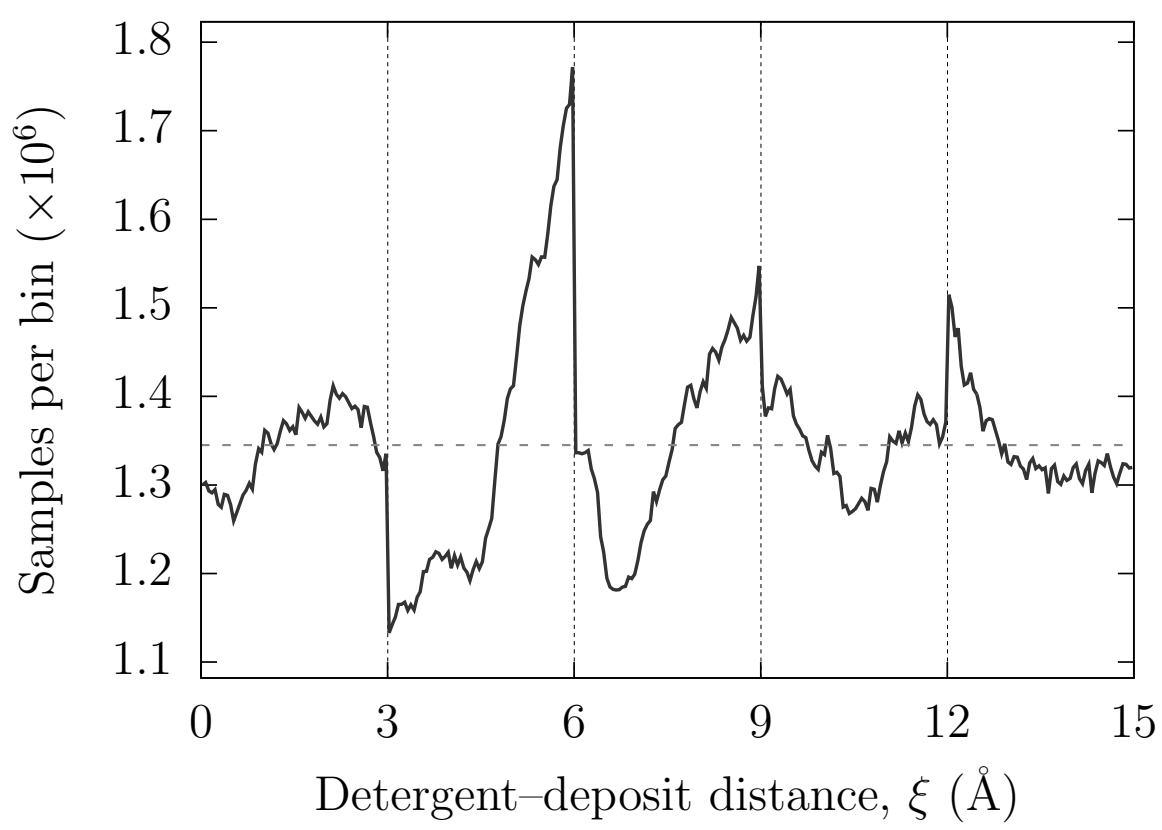

(a) Betaine DCA

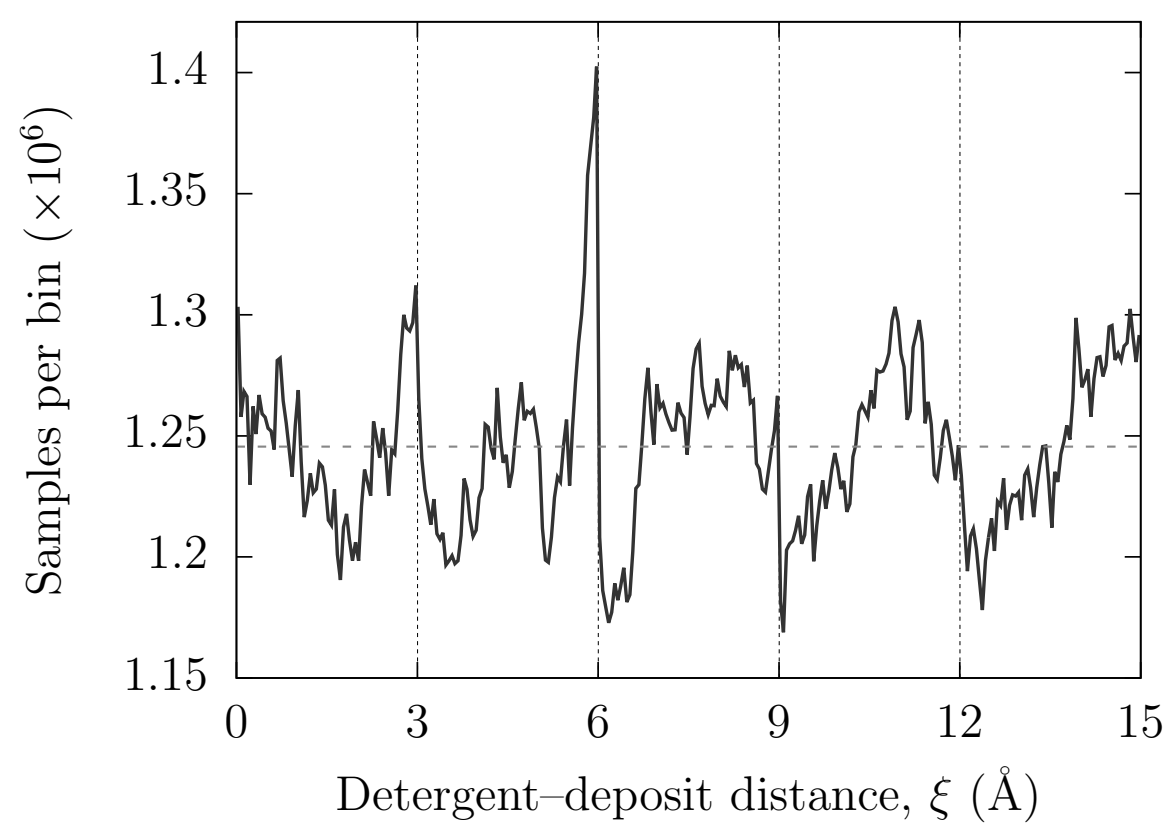

(b) Mannich base DCA

Figure S1: Bin-sampling histograms at E15 for (a) the betaine and (b) the Mannich base detergent. The adaptive bias drives the occupancy toward uniformity (uniform to within a factor $\sim$ 1.5; dashed line, mean). The residual peaks coincide with the 3 Å window boundaries (dotted) and are the harmonic wall artifact discussed at the end of this section.

The second diagnostic presented in Figure S2 is the convergence of the mean force, which is the quantity actually integrated. The force in each bin is a running average of the instantaneous force, so its statistical error according to a $t^{-1/2}$ law once the sample count n grows in proportion to the elapsed time.[1] The figures plots the root mean squared deviation (RMSD) of the running mean force from its final value against simulation time on a double logarithmic plot. For both DCAs, each of the five independent windows tracks the $t^{-1/2}$ law, with overall fitted slopes of -0.52 for betaine and -0.53 for the Mannich base. This power law decay is the signature of statistics-limited convergence, i.e. the estimate is bounded only by the amount of sampling, and the degrees of freedom orthogonal to ξ are equilibrating as required. A window whose force instead levels off, departing from this slope,

would signal quasi-nonergodic sampling and an unreliable force, thus invalidating the PMF stemming from it. The deviation drops below the $t^{-1/2}$ line at the longest times because it is referenced to each run own final mean force rather than to the (unknown) infinite-time limit. As t approaches the total run length, the running and reference estimates are built from almost the same data, so their difference is driven to zero faster than any power law. This is an artifact of the reference and carries no physical meaning, and as such the slopes reported above are fitted over the intermediate regime, where the reference is effectively independent.

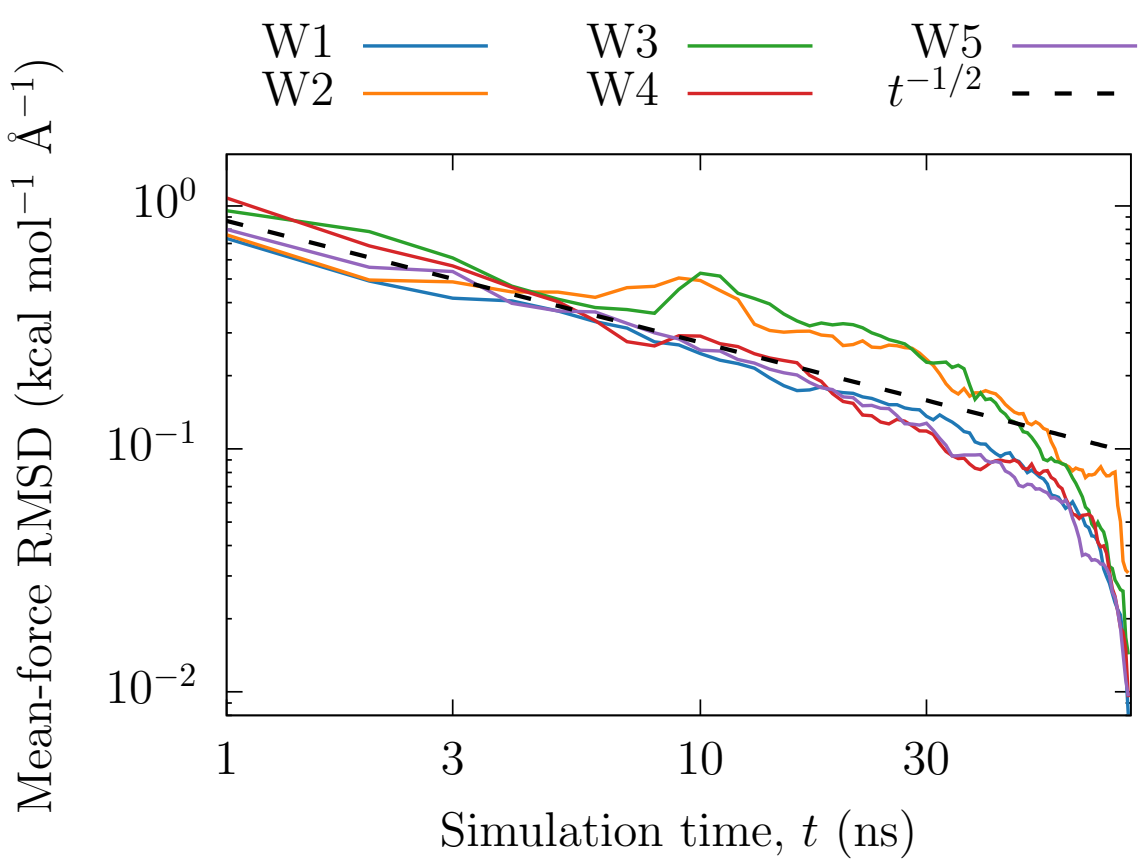

(a) Betaine DCA

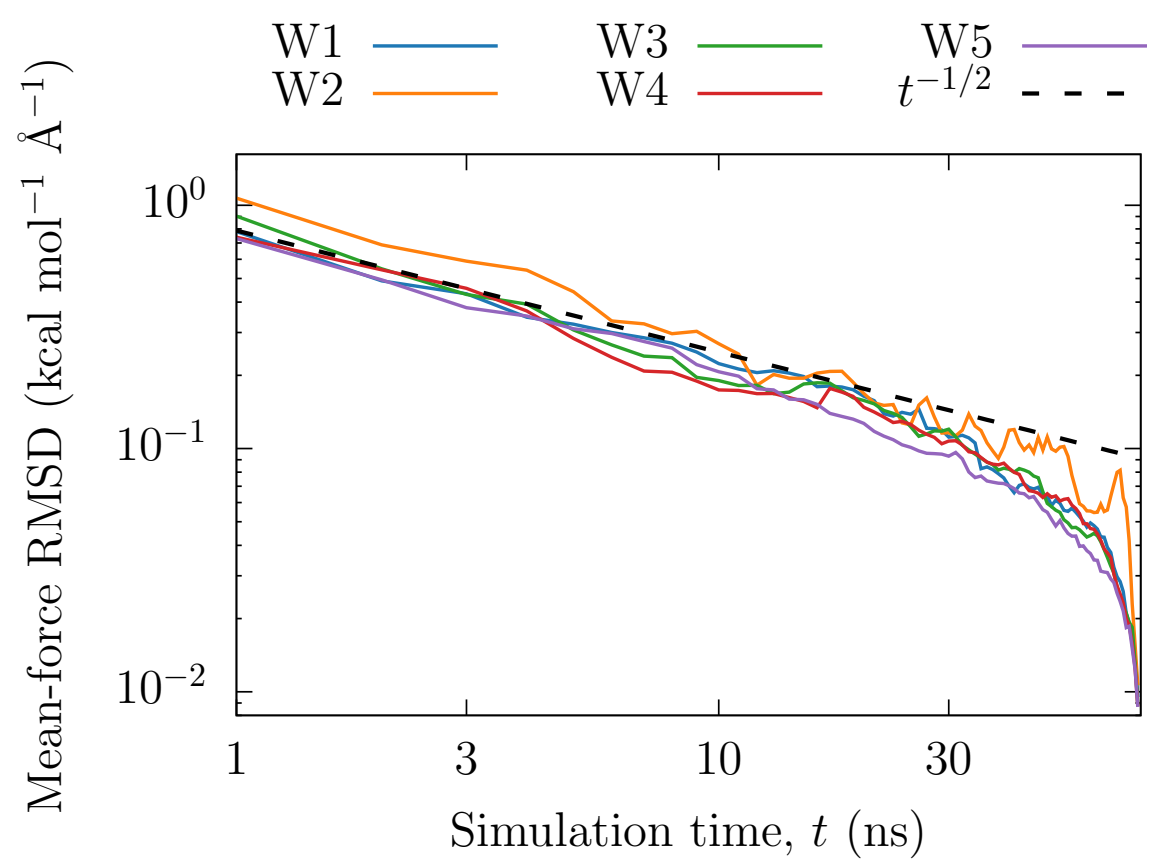

(b) Mannich base DCA

Figure S2: Convergence of the ABF mean force at E15: RMSD of the running mean force from its converged value against simulation time (logarithmic axes), for the five windows W1–W5 (coloured) compared with the $t^{-1/2}$ statistical law (black dashed line).

The third diagnostic is the continuity of the mean force across the window boundaries, which is presented in Figure S3 where the colour code for the different windows follows the convention of Figure S2. Because the windows are sampled independently, their forces are under no constraint to agree, so if they nonetheless join smoothly at the shared boundaries is an independent test for testing the PMF convergence. If, otherwise, a gradient discontinuity beyond the statistical error is found, then this may be a symptom of inadequate ergodic sampling. Away from the immediate window edges, as we discuss below, the consecutive windows meet to within $\sim 0.01\,\mathrm{kcal\,mol^{-1}\,\AA^{-1}}$, well within the per-bin noise and confirming that the independent windows sample a common ensemble.

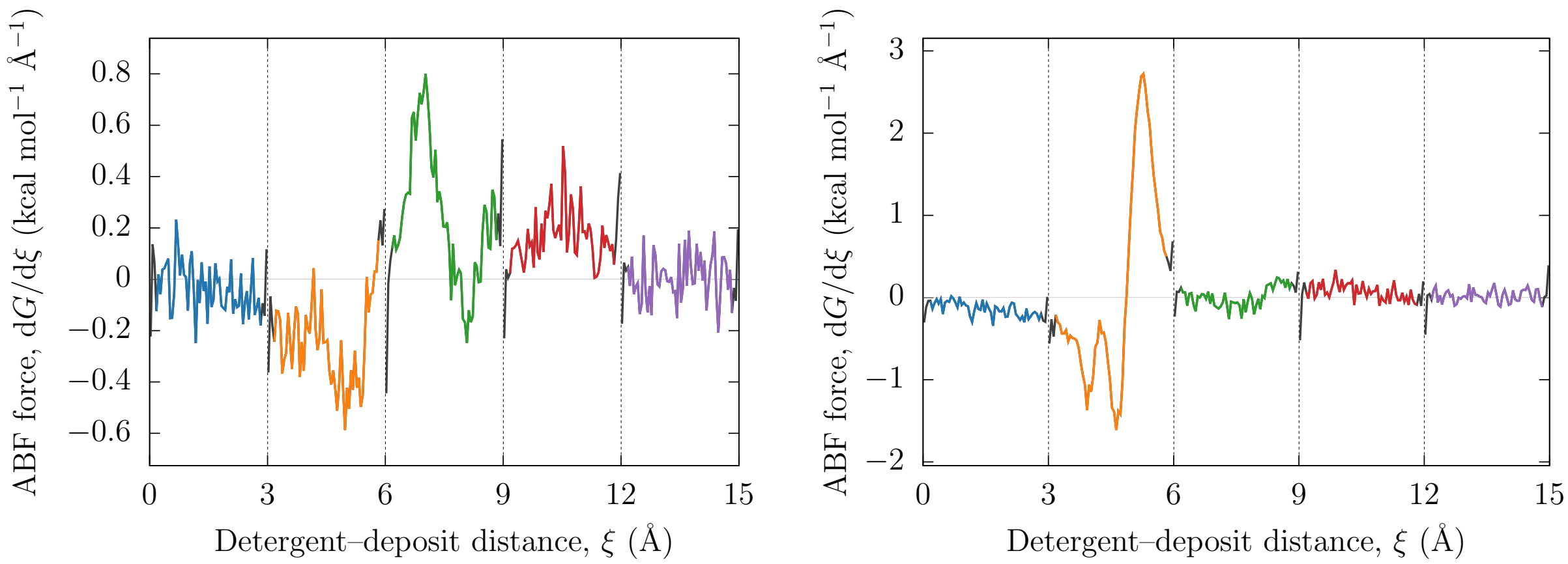

(a) Betaine DCA (b) Mannich base DCA

Figure S3: Converged mean force against ξ at E15. The mean force is the local gradient of the free energy profile, and as such it scatters about zero where the PMF is flat (the bulk and the plateau outside the contact region) and forms pronounced troughs and peaks where the PMF is steep (the descent into the adsorption well and the desolvation barrier beyond it). The coloured curves are the converged window interiors, which join continuously across the shared boundaries (vertical dotted lines). The full per-window force is overlaid in dark grey, and the one or two bins immediately adjacent to each boundary, where the grey departs from the interior, are the localised harmonic wall artifact.

The fourth diagnostic follows the assembled PMF itself as sampling accumulates, see Figure S4. The full profile, reconstructed by joining the per-window forces and integrating, is plotted at a sequence of increasing simulation times. The early estimates are visibly scattered but collapse onto a common curve well before the end of the run, after which the adsorption well depth no longer evolves and is indistinguishable from the production profile. The final and most demanding test is reproducibility across statistically independent trajectories, which also yields the most complete error estimate because it senses slow degrees of freedom that any single run may miss, see Figure S5. The stitched PMFs of the independent runs (three for each DCA) agree in the position of the adsorption well and in its depth to within $0.4\,\mathrm{kcal\,mol^{-1}}$, which we adopt as the reproducibility of a single PMF. For the zwitterionic DCA, the profiles differ on the short distance side of the well; nonetheless, this region does not enter the definition of ΔG_{ads}. The deeper and closer adsorption well of the Mannich base ($\Delta G_{\mathrm{ads}} \approx -2.0\,\mathrm{kcal\,mol^{-1}}$ at $\xi \approx 4.85\,\text{Å}$, against $\Delta G_{\mathrm{ads}} \approx -0.9\,\mathrm{kcal\,mol^{-1}}$

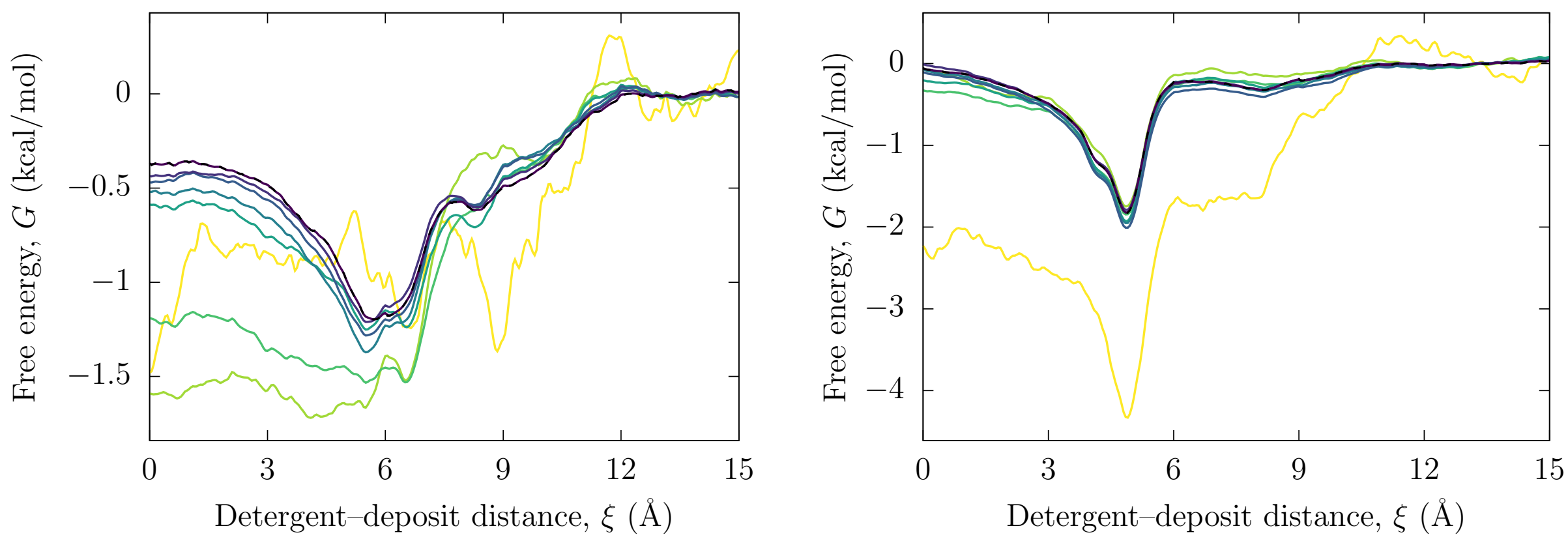

(a) Betaine DCA (b) Mannich base DCA

Figure S4: Stitched PMF reconstructed at increasing simulation times at E15. The shading runs from light (early simulation times) to dark (late simulation times), in steps of around 10 ns (i.e. yellow colour represents the free energy profile at 1 ns, and then at 11, 21, 31, etc. with darker colours). MD simulations have been run long enough for the ABF algorithm.

at $\xi \approx 5.5$ Å for betaine) is as well converged as the weaker one, and the stronger adsorption of the Mannich base reported in the main text rests on equally robust free energy estimates. We do not attach within-run statistical error bars to the PMF. The analytical estimate of the integrated error requires the autocorrelation time of the instantaneous force,[1] which the interval of our force output in COLVARS[2] is too coarse to resolve. The agreement between independent runs supplies the uncertainity instead, and as an average over entire trajectories it is even a more conservative measure.

The only systematic departures from uniform sampling occur at the window boundaries, which can be interpreted as the residual peaks in the sampling histograms (Figure S1) and as the spikes confined to the outermost bins of each window in the continuity plots (Figure S3). Both represent the same consequence of the harmonic walls that hold each window in place. A wall of finite stifness k allows the coordinate to penetrate a short distance, of order $(k_B/k)^{1/2} \approx 0.1$ Å or one-to-two bins, and to dwell at the turning point. This enhances the local occupancy and contaminates the force estimates in those edge bins with the restraint contribution. ABF cannot remove this enhancement because the wall is an externally imposed harmonic potential and not part of the free energy surface that the

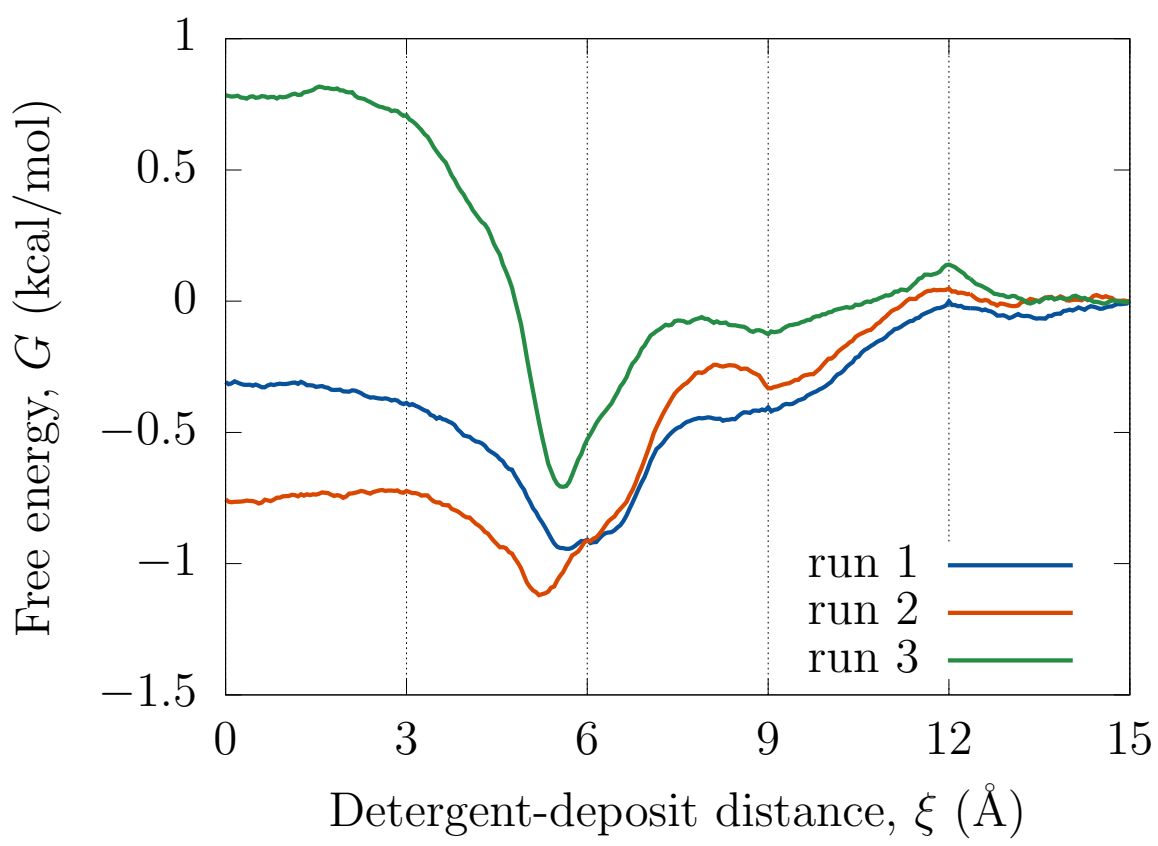

(a) Betaine DCA

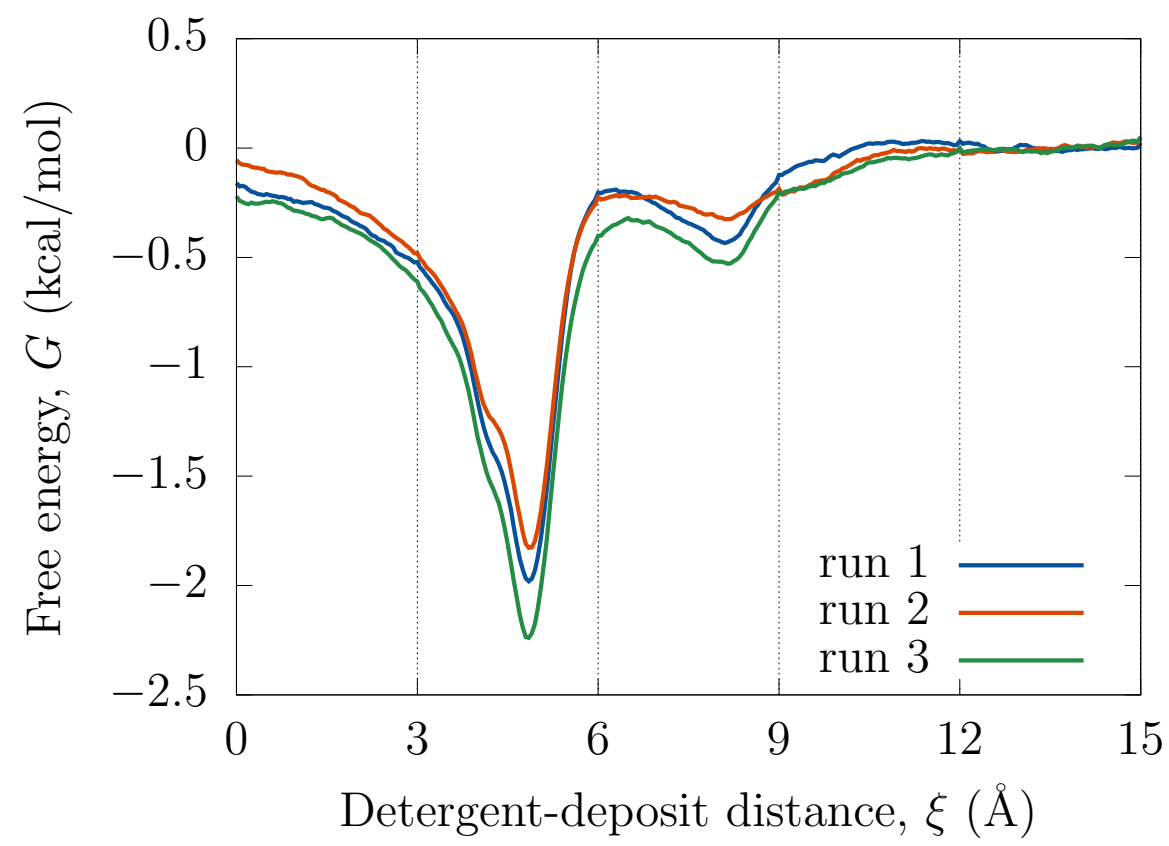

(b) Mannich base DCA

Figure S5: Reproducibility across statistically independent runs. Full reconstruction of the PMFs for independent runs at E15, coming from different trajectories. The profiles agree in the position and depth of the adsorption well to within the run-to-run scatter. Because such trajectories explore the slow degrees of freedom, orthogonal to ξ, this agreement is the strongest convegence check available, and the spread between runs sets the uncertainty we quote on the adsorption energy ΔG_{ads}.

bias learns. This effect is strictly local: the window interiors are completely unaffected, and because a bias of $\sim 0.5\,\text{kcal}\,\text{mol}^{-1}\,\text{Å}^{-1}$ confined to a single 0.05 Å bin integrates to a negligible step, it does not propagate into the PMF. Indeed, the profile obtained by integrating the concatenated per-window forces reproduces the production profile to $0.002\,\text{kcal}\,\text{mol}^{-1}$. The boundary peaks are thus an expected feature of the stratification protocol rather than a sign of poor convergence.

Taken together, these five diagnostics are not an arbitrary collection of checks but trace the chain of dependencies on which an ABF calculation is based. The PMF is reconstructed by integrating an estimated mean force, so its reliability depends on the sampling that feeds the force, on the convergence of the force itself, on the consistency of the windowing used to accelerate that convergence, on the stationarity of the integrated profile, and finally on its reproducibility. We show that the adsorption PMFs are converged, which is what ultimately licences the conclusions of the main text: the mechanistic comparisons we draw from these free energies, between the zwitterionic and the Mannich detergent and across ethanol content, rest on free energy differences that are well resolved, and so reflect genuine differences in the

physical chemistry of the DCA-solvent system rather than residual sampling noise.

2 PMF studies for PIBSI within gasoline surrogates

The free energy profile for the adsorption of PIBSI on circumovalene within gasoline surrogates is presented in Figure S6. In contraposition to the zwitterionic DCA binding on circumovalene shown in the main text, the adsorption process is not as affected when increasing the concentration of polar species in the base solvent. This aligns with the fact that the PIBSI headgroup, being a non-ionic particle, would have little preference for one solvent over the other.[3] This poses an interesting scenario because even for very small biofuel con-

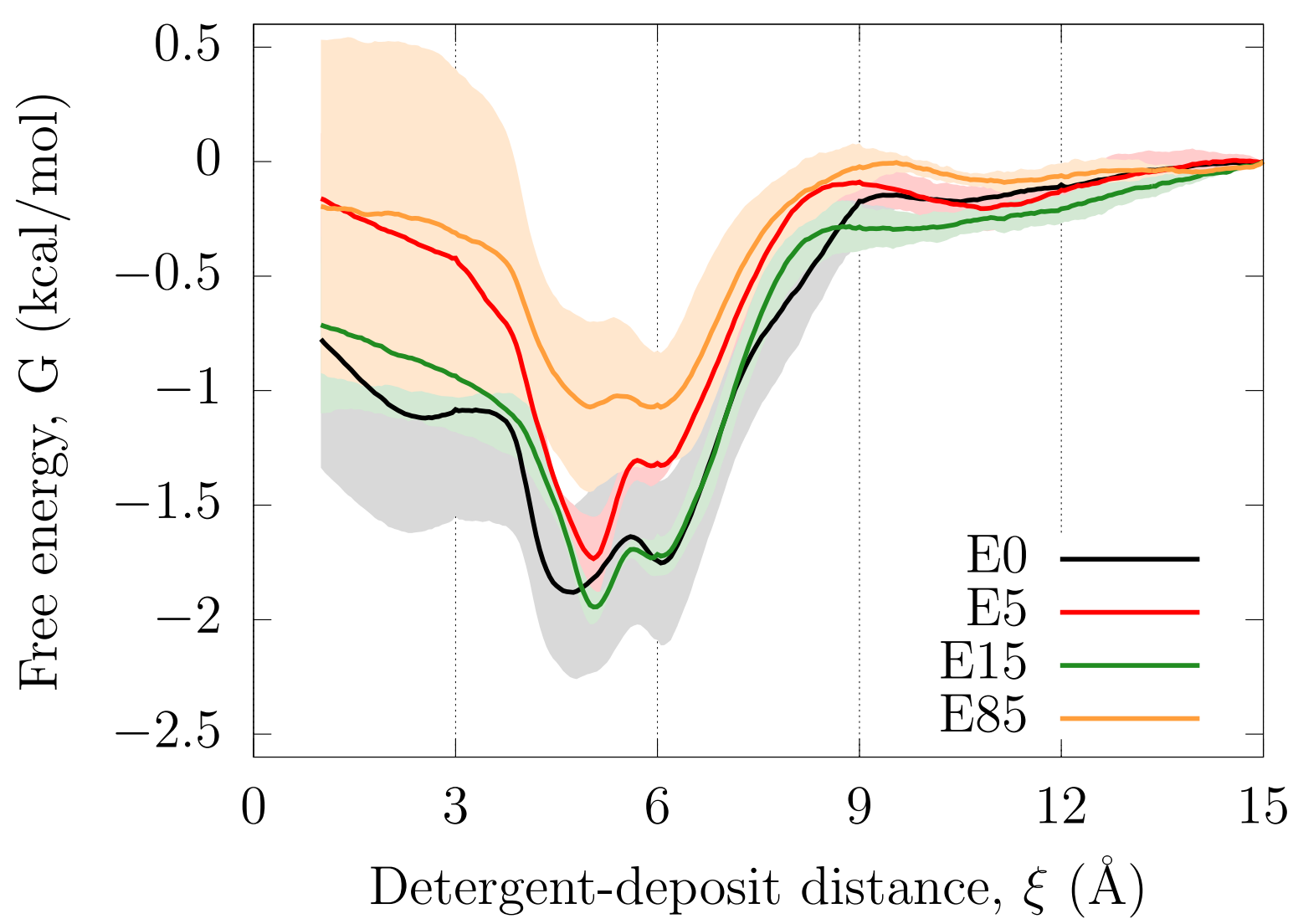

Figure S6: Adsorption PMF for the PIBSI detergent from biofuel mixtures with different ethanol concentrations. For this DCA chemistry, the adsorption physics are only affected for large concentration of the polar species.

centrations (E5) the zwitterionic DCA loses its advantage, $\Delta G_{\text{ads}} = (-1.37 \pm 0.11)\,\text{kcal}\,\text{mol}^{-1}$ versus $\Delta G_{\text{ads}} = (-1.74 \pm 0.10)\,\text{kcal}\,\text{mol}^{-1}$ for PIBSI, although PIBSI adsorption is roughly three times weaker than that of the zwitterionic DCA in pure iso-octane. From E15 onwards, PIBSI binds more strongly (see Table 1 in the main text). At intermediate concentrations (e.g. E15), the PMF is still not affected by the presence of polar species in the base solvent,

$\Delta G_{\text{ads}} = (-1.95 \pm 0.04)\,\text{kcal}\,\text{mol}^{-1}$. At high ethanol concentrations (e.g. E85), however, the adsorption strength is roughly halved, $\Delta G_{\text{ads}} = (-1.15 \pm 0.17)\,\text{kcal}\,\text{mol}^{-1}$, despite being a non-ionic surfactant, although it still binds significantly more strongly than the zwitterionic DCA, $\Delta G_{\text{ads}} = (-0.39 \pm 0.11)\,\text{kcal}\,\text{mol}^{-1}$, whose adsorption free energy at E85 is not statistically distinguishable from zero. It is still not clear why there is this decrease in performance for the PIBSI formulation for large ethanol concentrations, since it does not happen with the Mannich base, $\Delta G_{\text{ads}} = (-2.14 \pm 0.03)\,\text{kcal}\,\text{mol}^{-1}$ as in Figure 8 in the main text. Some mechanistic conjectures are proposed in the discussion that follows, aligning with the PMF results.

In the case of gasoline surrogates, with ethanol as the biofuel, the spatial development of alcohol-based networks is not related to the PIBSI dynamics. A similar rationale applies to the Mannich base formulation, for which it was found an apparent free energy independence with ethanol concentration, although the actual picture is more nuanced if one pays attention to the enthalpy-entropy decomposition. The further drop in free energy found at E85 could be ascribed to a concentration-dependent formation of hydrogen bonds between PIBSI and ethanol. The more hydrophilic part of PIBSI is its polyamine headgroup, composed of three secondary amines and one primary amine in the terminal group. As shown later in this Supporting Information document, the fundamental interactions of both ethanol and iso-octane at the DFT level of theory with secondary amines (in the Mannich base formulation) show that either solvent is expected to be found near a $-$NH$-$ functional group.

However, with E85 formulations, there are enough alcohol molecules in the solvent that might not engage in ethanol clusters, in particular next to the PAH deposit. This spare ethanol molecules can then be subjected to the next set of more favourable interactions. Around the PIBSI detergent, these will be the formation of hydrogen bonds with the secondary amines, where the O atoms in the alcohol behave as acceptors. It should be mentioned that hydrogen bonds where amines act as the donors are weaker than those related with alcohols, since N is a less electronegative atom than O, and therefore its dipole moment is

lower. This picture might explain why at lower ethanol concentrations there is no change in the PIBSI adsorption on PAH, as the number of ethanol molecules is small enough so that all of them gather in the form of clusters without being disrupted by other elements. This sort of implicitly captures the transition from an enthalpy-driven adsorption process at low biofuel concentrations to entropy-dominated at larger concentrations. For the Mannich base, this is not necessarily happening at E85 because the ethanol molecules can engage in interactions with other functional moieties in the system (aromatic ring, −OH group, etc.), without disrupting the sheer interactions of the polyamine headgroup.

3 Zwitterion-solvent DFT calculations

The zwitterionic head-group poses a particularly complicated problem in DFT terms because of the many conformations the molecule might adopt and the number of heteroatoms. Therefore, the free energy landscape based on the electronic degrees of freedom will consist on a large number of metastable states. Depending on the initial configuration used in the DFT unit cell (i.e. on the relative location of the solvent molecule with respect to the DCA), the output from the ionic relaxation process will be different, with no guarantee of finding the global minimum. In the field of computational drug studies, there are streamlined ways to accomplish this task, where the process is known as docking. This is motivated by the important problem of predicting the preferred orientation of one molecule with respect to another when both are bound to each other forming a stable complex. Here we are not interested in unequivocally finding the global minimum, but to get a flavour of which physical interactions are the most important ones from a thermodynamic standpoint. Therefore, we run independent DFT simulations at the same level of theory using different initial configurations, and compare the relative free energies predicted for each kind of interaction.

We exemplify these intricacies by introducing studies of how the different solvent molecules in the gasoline surrogate (namely, ethanol and iso-octane) interact with the zwitterionic head-

group. This detergent-solvent analysis would permit to understand which solvent species are prone to interact with the detergent, providing valuable context to understand the PMF results yielded by ABF-MD simulations. The more active solvent species (e.g. ethanol, owing to its larger dipole moment) is taken as the reference for carrying this study, which will then be mimicked with iso-octane to understand the relative interaction energies. Six different positions in the zwitterionic head-group are identified as more likely to interact with the solvent, presented in Figure S7. In all cases, the interaction energy E_{int} is computed in

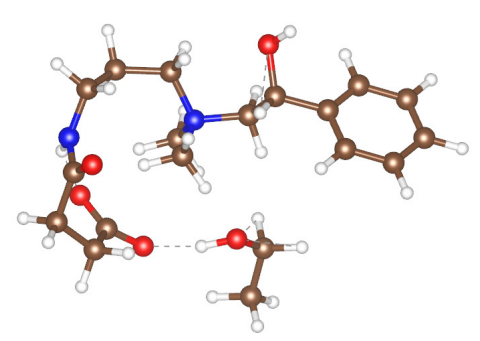

(a) $E_{\mathrm{int}} = -20.9\,\mathrm{kcal\,mol^{-1}}$

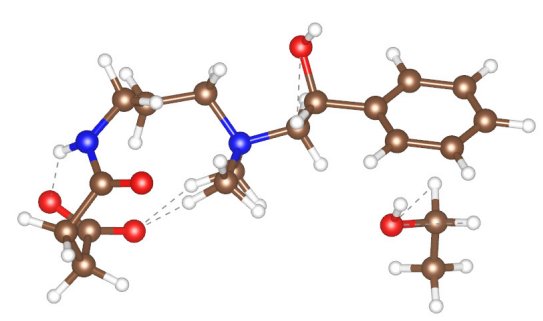

(b) $E_{\mathrm{int}} = -7.9\,\mathrm{kcal\,mol^{-1}}$

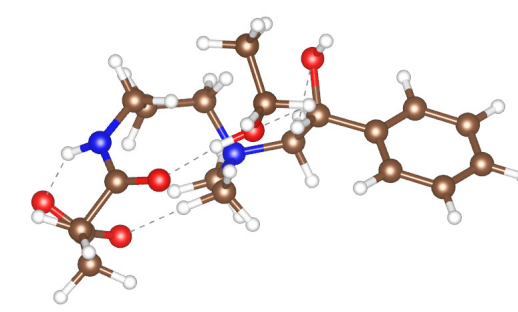

(c) $E_{\mathrm{int}} = -17.7\,\mathrm{kcal\,mol^{-1}}$

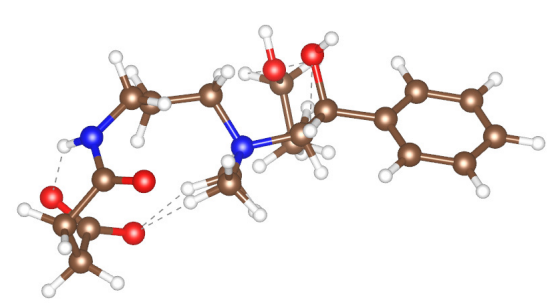

(d) $E_{\mathrm{int}} = -6.9\,\mathrm{kcal\,mol^{-1}}$

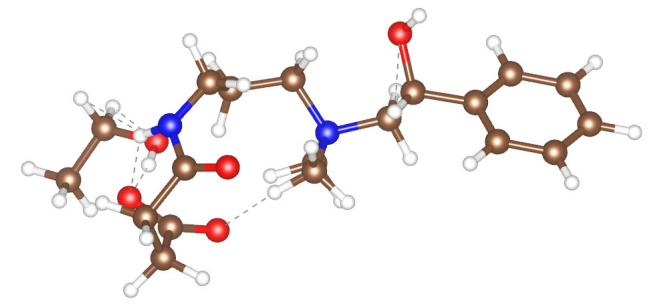

(e) $E_{\mathrm{int}} = -16.0\,\mathrm{kcal\,mol^{-1}}$

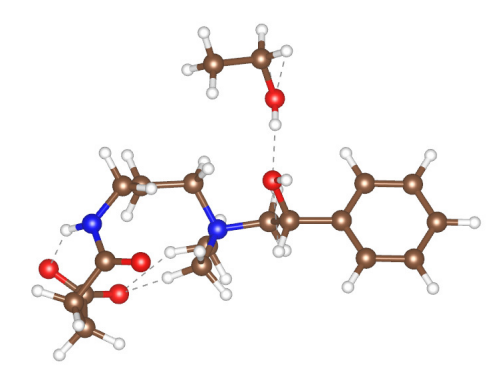

(f) $E_{\mathrm{int}} = -9.6\,\mathrm{kcal\,mol^{-1}}$

Figure S7: Zwitterion - ethanol interactions after ionic relaxation for different binding sites in the zwitterionic head-group.

vacuum according to

$$E_{\mathrm{int}} = E_{\mathrm{sys}} - (E_{\mathrm{sol}} + E_{\mathrm{DCA}})\,, \tag{1}$$

where E_{sys} is the total energy of the system comprising both DCA and solvent after the geometry relaxation process, and E_{sol} and E_{DCA} are the gas-phase energies of the geometrically relaxed solvent molecule and zwitterionic head-group, respectively. We can see that out of the six configurations considered in this study, three are more energetically favourable than the others: Figure S7a, Figure S7c, and Figure S7e. In the first case, the strong interaction energy arises from the combination of i) a hydrogen bond between ethanol and one O from the carboxylate anion and ii) the ion-dipole interaction between the cation and

O in the ethanol hydroxyl motif. In the second case, the interaction energy arises from the combination of i) a weaker hydrogen bond between ethanol and O in the amide group (O−H· · · O) and ii) the aforementioned ion-dipole interaction. In the third case, there is a strongly directed hydrogen bond between ethanol and one O belonging to the carboxylate anion.

Equivalent geometries for these three strongly interacting cases that probe the interactions between the zwitterionic head-group and iso-octane are presented in Figure S8. Despite

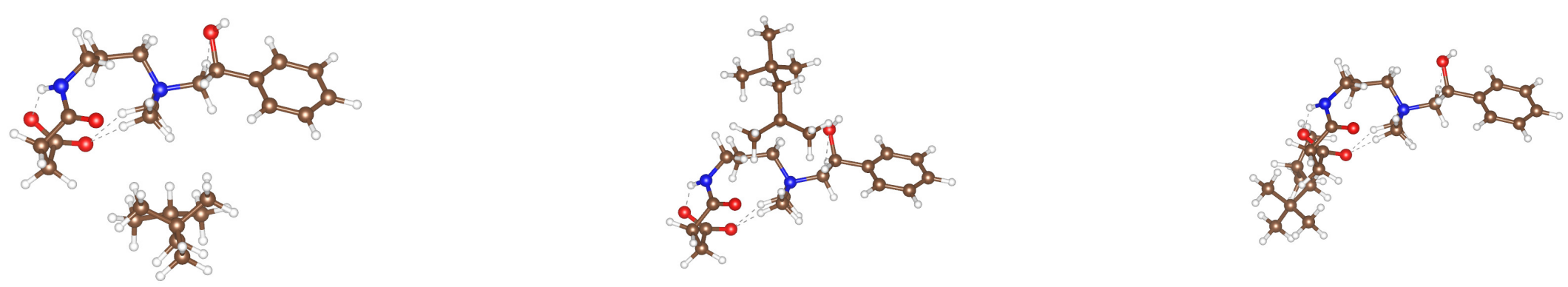

(a) $E_{\text{int}} = -8.5\,\text{kcal}\,\text{mol}^{-1}$ (b) $E_{\text{int}} = -8.5\,\text{kcal}\,\text{mol}^{-1}$ (c) $E_{\text{int}} = -6.8\,\text{kcal}\,\text{mol}^{-1}$

Figure S8: Zwitterion - octane interactions after ionic relaxation for different binding sites in the zwitterionic head-group.

the iso-octane is placed in geometrically similar positions as to ethanol, there is an obvious tendency in that interaction energies are much lower for the non-polar species. This clarifies that when the zwitterionic DCA interacts with a solvent composed of ethanol and iso-octane, the more polar species will tend to be more strongly attracted to the detergent. As such, ethanol is preferentially concentrated next to the DCA molecule, and the formation of clusters is locally concentrated in this region. The scaling between these gas-phase interactions and the ones in the liquid environment is studied in the main text.

4 Structural studies of the solvent in biofuel surrogates

We show the intensive self-network RDFs for both betaine and Mannich systems in Figure S9. The main plots capture the O−O RDF pair for ethanol at different concentrations, whereas the insets reflect the C−C RDF pairs for iso-octane. The produced $g(r)\rho$ show that, from E5 to E15, ethanol forms reverse micelles within the non-polar iso-octane bulk. The polar

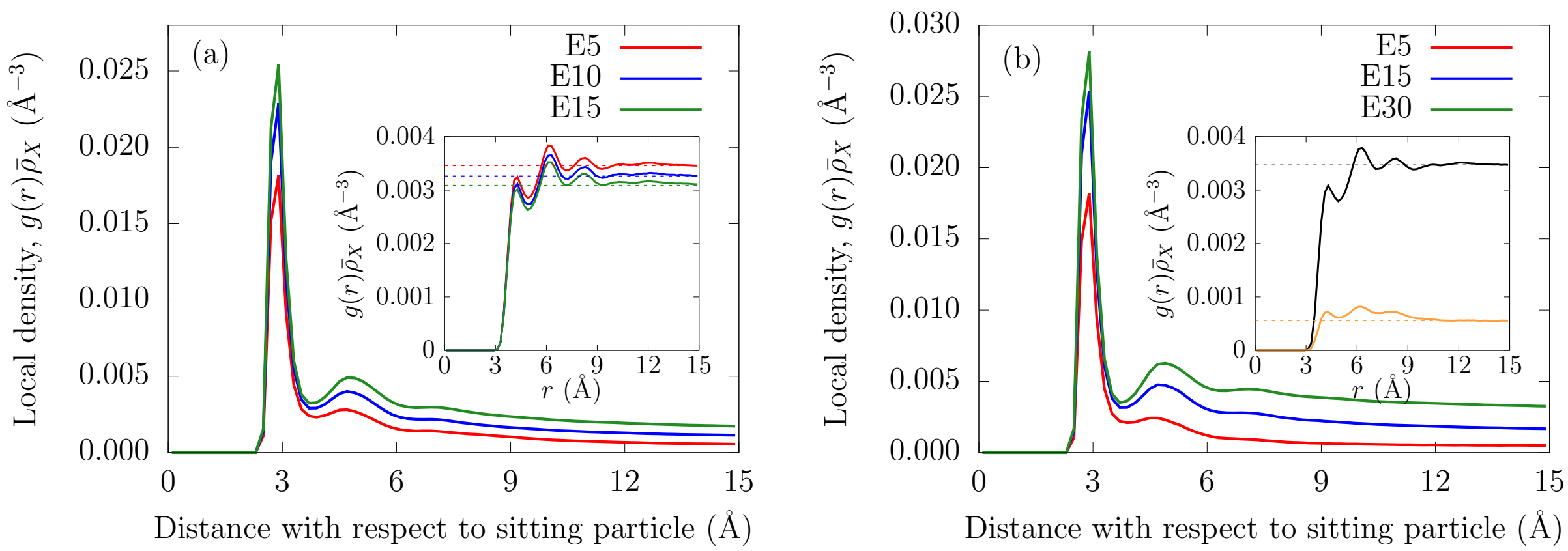

Figure S9: Local number density for the self-network O-O pairs at different ethanol concentrations for (a) betaine DCA and (b) Mannich base DCA systems. Insets show the C-C pairs for the carbons in methyl groups in iso-octane. Black line represents E0, orange line represents E85.

−OH groups point towards the center of the micelle and undergo hydrogen bonds with each other, whereas the short alkyl chain points outwards due to van der Waals interactions with the iso-octane molecules. The distance at which the main O-O peaks (2.9 Å) occur agrees well with the equilibrium distance coming from the DFT geometry relaxation (2.8 Å) of two ethanol molecules in vacuum. The C-C RDF for $-CH_3$ groups within iso-octane is presented in the insets, having a first peak at around 4.2 Å and the main peak at around 6.3 Å. The iso-octane RDFs also agree well with the DFT-optimised dimer geometries, where the closest C−C pairs start at 4.1 Å, whereas the bulk of such distances cluster at 5.5−6.3 Å, in good agreement with the better developed second peak.

The inverse micelle structuring found in MD simulations is consistent with the solvent-solvent interactions from our DFT calculations. The hydrogen bond-mediated interactions between ethanol molecules ($E_{\text{int}} = -6.9\,\text{kcal}\,\text{mol}^{-1}$) is favoured compared to the dipole-induced dipole interactions between ethanol and iso-octane ($E_{\text{int}} = -3.2\,\text{kcal}\,\text{mol}^{-1}$). The strength of the ethanol-ethanol hydrogen bond agrees well with values from first-principles calculations with higher levels of theory and experiments,[4] which supports the accuracy of the non-local functional used during this study. Furthermore, the dispersion-based interaction

between iso-octane molecules ($E_{\mathrm{int}} = -4.5\,\mathrm{kcal\,mol^{-1}}$) is stronger than the dipole-induced dipole O···CH interaction between dissimilar solvent species.

5 Mannich-solvent DFT calculations

When a similar DFT study is carried out with the Mannich base headgroup, no obvious interaction energy differences are found between ethanol and iso-octane. Thus, the picture of the detergent acting as an "aggregation point" for the formation of solvation shells, as in the zwitterionic DCA, is no longer supported. Indeed, as shown in the main text, the fluid structure produced in MD simulations is not affected by the presence of the detergent, without a clearly defined cybotactic region surrounding the solute.

The more relevant Mannich-solvent DFT interactions are presented in Figure S10, with respect to both ethanol and iso-octane, as a function of different non-covalent interactions. Different configurations are considered by targeting the more reactive motifs in the Mannich base formulation: the aromatic ring (first column), the secondary amine next to it (second column), and the tertiary amine in the tip of the headgroup (third column). The strongest

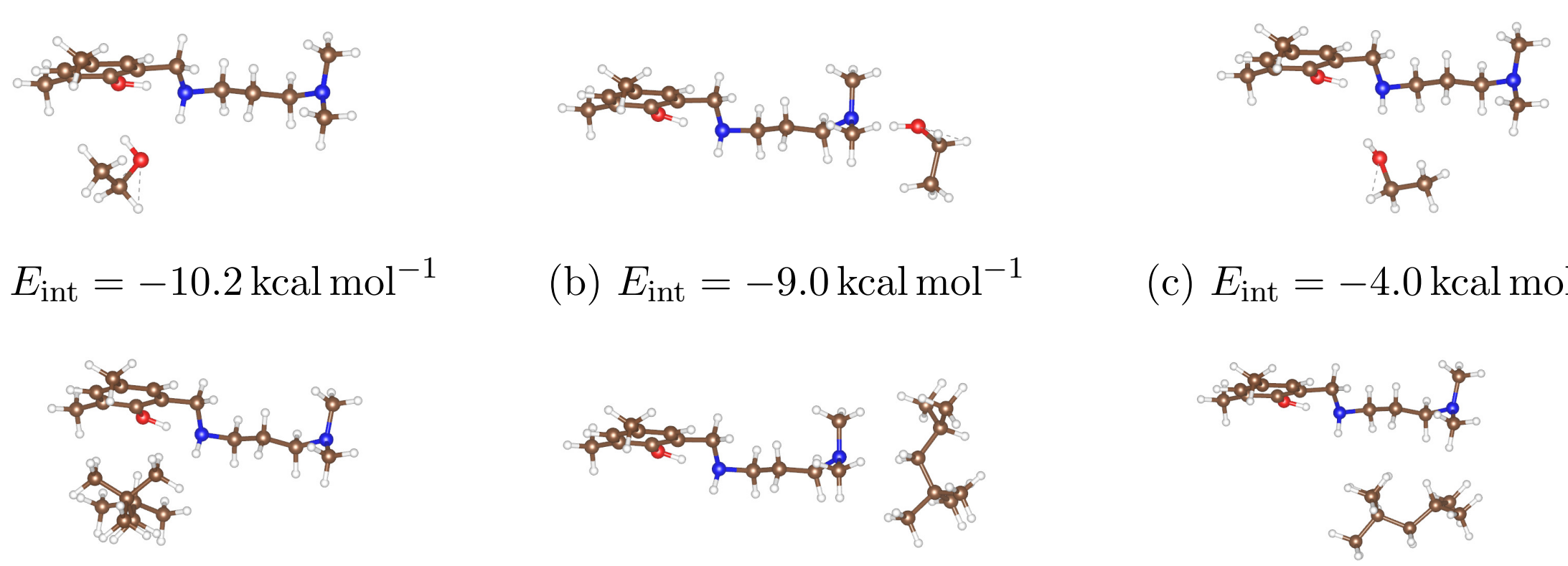

(a) $E_{\mathrm{int}} = -10.2\,\mathrm{kcal\,mol^{-1}}$ (b) $E_{\mathrm{int}} = -9.0\,\mathrm{kcal\,mol^{-1}}$ (c) $E_{\mathrm{int}} = -4.0\,\mathrm{kcal\,mol^{-1}}$

(d) $E_{\mathrm{int}} = -10.1\,\mathrm{kcal\,mol^{-1}}$ (e) $E_{\mathrm{int}} = -2.3\,\mathrm{kcal\,mol^{-1}}$ (f) $E_{\mathrm{int}} = -5.2\,\mathrm{kcal\,mol^{-1}}$

Figure S10: Mannich - solvent interactions after ionic relaxation for different binding sites in the Mannich head-group. Top row shows the optimal conformations for ethanol, whereas bottom row shows the interactions with iso-octane.

of interactions for the Mannich DCA occurs with ethanol interacting with the aromatic ring,

see Figure S10a, where three hydrogen bonds are formed. The hydroxyl group in ethanol, the hydroxyl group attached to the aromatic ring, and the secondary amine all work as hydrogen bond donors and acceptors in a stable triangular synergetic configuration. On the other hand, iso-octane interacts with the aromatic ring through CH$\cdots\pi$ interactions, with very similar energetics in Figure S10d. Interactions of either solvent species with either the tertiary or the secondary amine result in less stable configurations, although the key message is that no ethanol or iso-octane is preferred to interact with the Mannich base. This different mechanistic behaviour underpins the better performance of the Mannich base in alcohol-containing gasoline surrogates.

6 Mannich base DCA: PMF at different temperatures

From a conceptual point of view, the adsorption process from a solution is better framed not simply as the attraction between the adsorbent (DCA) and the adsorbate (PAH deposit), but as an exchange in which DCA–solvent and deposit–solvent contacts are replaced by direct DCA–deposit contacts, with the displaced solvent returning to the bulk. The enthalpy and entropy of adsorption are governed by the interacting surfactant, the surface that is contacted, and by the solvent that is displaced. It is worth noting that all of these elements are expected to change character as the solvent polarity is varied with ethanol content.

In the weakly polar iso-octane rich solvent (characterised by e.g. E15), the solvent is unstructured and interacts with the detergent and the deposit only through weak dispersion forces. Stripping iso-octane from the PAH surface costs little enthalpy and the displaced molecules, already disordered in the bulk, gain negligible entropy on the PAH release. DCA adsorption is then dominated by the favourable enthalpy from the non-covalent interactions (e.g. $\pi-$stacking, dispersion contacts) arising between the Mannich base head-group and the PAH deposit, so that an enthalpy-driven process with $\Delta H_{\text{ads}} < 0$ and $\Delta S_{\text{ads}} < 0$ is expected.

On the other hand, in the strongly polar ethanol rich solvent (characterised by e.g. E85),

ethanol cannot undergo hydrogen bonds with the hydrophobic deposit or to the non-polar regions of the DCA head-group. Instead, ethanol molecules adopt ordered self-configurations around these groups to preserve its own thermodynamically favourable network. This ordered solvation shell is analogue to the hydrophobic hydration of non-polar solutes in aqueous systems.[5,6] Upon adsorption, the hydrophobic DCA motifs and the PAH deposit come together and release the ordered ethanol into the disordered bulk, so the process is driven by the resulting gain in solvent entropy. The loss of the favourable shell interactions renders the enthalpy unfavourable, giving an entropy-driven process with $\Delta S_{\mathrm{ads}} > 0$ and $\Delta H_{\mathrm{ads}} > 0$. This is an expectation about the direction of the change; whether the entropic term becomes dominant, or simply less unfavourable than at low ethanol content, has to be determined from the PMF temperature dependence.

The adsorption free energy of the Mannich base DCA is found to be almost independent of ethanol concentration in the main text, see Section 3.2.3. The free energy alone is a poor probe of the change in the thermodynamics of adsorption, because the enthalpic and the entropic contributions can vary in opposite directions while their sum, $\Delta G_{\mathrm{ads}} = \Delta H_{\mathrm{ads}} - T\Delta S_{\mathrm{ads}}$, remains constant. A nearly ethanol-independent ΔG_{ads} is consistent with either an unchanged adsorption mechanism or with a compensating shift between enthalpy- and entropy-driven binding. To distinguish between these scenarios, we decompose the adsorption free energy into its enthalpic and entropic parts through the PMF temperature dependence, following the finite-difference approximation introduced in Eq. 3 of the main text. Since the binding free energy obeys $d\Delta G_{\mathrm{ads}}/dT = -\Delta S_{\mathrm{ads}}$, the temperature dependence reports the sign of the adsorption entropy directly: a process opposed by entropy ($\Delta S_{\mathrm{ads}} < 0$) becomes weaker with increasing temperature, whereas one driven by entropy ($\Delta S_{\mathrm{ads}} > 0$) strengthens.

For the two representative concentrations of low and high ethanol content, E15 and E85, we compute the adsorption PMF at $T = 300$, 325, and 350 K, with three statistically independent ABF-MD runs at each temperature, started from different initial velocities.

Every profile is referenced to $G(\xi = 15$ Å$) = 0$, and ΔG_{ads} is read at the PMF minimum, which falls at $\xi = 4.85$ Å for all 18 runs. Throughout, the statistical unit is the independent run: we report means over the three runs and quote as uncertainty the standard error of the mean, $\text{SE} = \text{SD}/\sqrt{3}$, where SD is the run-to-run standard deviation. The SD is the reproducibility of a single simulation and is what the shaded bands show in Figs. S11, S12, and 13; for Fig. 13 it is propagated as $\sigma(-T\Delta S) = 6.5\,[\text{SD}^2_{300} + \text{SD}^2_{350}]^{1/2}$ and $\sigma(\Delta H) = [\text{SD}^2_{325} + \sigma(-T\Delta S)^2]^{1/2}$, i.e. the band is the spread expected for a decomposition build from a single run per temperature.

Eq. 3 is evaluated at 325 K as the central difference between the run-averaged 300 K and 350 K profiles, $-T\Delta S = T\,[\bar{G}(350\,\text{K}) - \bar{G}(300\,\text{K})]/50\,\text{K}$. For three equally spaced temperatures, this is identical to the slope of a least-squares straight line fitted to G against T through all nine runs; $\Delta H = \bar{G}(325\,\text{K}) + T\Delta S$. Because the decomposition divides a free energy difference by a temperature interval, its statistical uncertainty is that of ΔG_{ads} amplified by $T/(2\Delta T) = 6.5$ (13 for a one-sided difference over 25 K), which is why independent runs are needed to resolve it. The statistical treatment follows from the nature of the data. Each ΔG_{ads} is an average over some 10^8 force samples of one trajectory, so the values obtained from independent runs at a given temperature scatter approximately normally about the ensemble mean, with a spread that is not known beforehand and has to be estimated from the same few runs. The run-to-run SDs at the six state points (0.05–0.31 $\text{kcal}\,\text{mol}^{-1}$) show no systematic dependence on temperature, so a common variance is assumed within each concentration.

The standard error of the fitted slope then follows from the scatter of the nine points about the line, and because that standard error is itself an estimate from few runs, the ratio $t = \text{slope}/\text{SE}$ follows the Student's t distribution rather than a normal one: the nine runs determine two parameters, intercept and slope, leaving 9-2=7 degrees of freedom, whose two-sided 5 % threshold is $|t| = 2.36$ instead of the normal 1.96, with 95 % intervals of $\pm 2.36\,\text{SE}$. The difference between the two concentrations is obtained from a single fit through

the 18 runs of both systems with an intercept and a slope for each concentration, leaving $18 - 4 = 14$ degrees of freedom (threshold $|t| = 2.14$). We report t together with the two-sided p-value, the probability of obtaining a slope at least as large in magnitude if ΔG_{ads} did not depend on temperature, and regard ΔS_{ads} as resolved when $p < 0.05$. Since $-T\Delta S_{\text{ads}} = T\,\mathrm{d}\Delta G_{\text{ads}}/\mathrm{d}T$, the same t and p apply to the entropic term, and the uncertainty of ΔH_{ads} combines those of $\Delta G_{\text{ads}}(325\,\text{K})$ and of the slope. All uncertainties are statistical; systematic contributions common to all runs (force field, finite size, the finite-difference approximation) are not included.

The resulting PMFs are shown in Figure S11 and the values at the minimum are collected in Table S1. At E15 the well depth grows steadily as the temperature is lowered, from $\Delta G_{\text{ads}} = -2.06 \pm 0.12$ kcal mol^{-1} at 350 K to -2.60 ± 0.10 at 325 K and -2.93 ± 0.12 at 300 K. The slope $d\Delta G/dT = -\Delta S_{\text{ads}} = -17.4 \pm 3.1$ cal mol^{-1} K^{-1} is resolved at $t = 5.6$ ($p = 0.0008$). At E85, the three profiles lie on top of one another within the run-to-run scatter (-2.14 ± 0.03, -2.07 ± 0.15, -1.92 ± 0.18 kcal mol^{-1} at 350, 325, and 300 K, respectively) and $\Delta S_{\text{ads}} = 4.3 \pm 3.6$ cal mol^{-1} K^{-1} is not resolved ($t = 1.2$, $p = 0.26$) with the 95% interval on $-T\Delta S$ from -4.2 to +1.3 kcal mol^{-1}. The difference between the two concentrations is resolved at $t = -4.6$ on 14 degrees of freedom ($p = 0.0004$). Equation 3 in the main text assumes that S is independent of temperature over the interval sampled, i.e. that $\Delta G(\xi, T)$ is linear in T, which the three temperatures allows us to test: the curvature ΔG(325 K)-0.5[ΔG(300) K+ΔG(350) K at the minimum is -0.10 ± 0.13 kcal mol^{-1} at E15 and -0.04 ± 0.18 kcal mol^{-1} at E85.

The values above refer to the PMF minimum, which is how ΔG_{ads} is defined in this work. Since a decomposition could in principle look different elsewhere along the profile, the same replica analysis is repeated at every bin of the collective variable (Figure S12). At E15, $\mathrm{d}G/\mathrm{d}T$ is positive and significant ($|t| > 2.36$, 7 degrees of freedom) from contact to $\xi \approx 11$ Å, i.e. across the contact well, the second solvent layer and the approach region, with t between 5 and 8 up to 8.5 Å. At E85 no bin is significant: the largest $|t|$ is 2.25, at 4.25 Å,

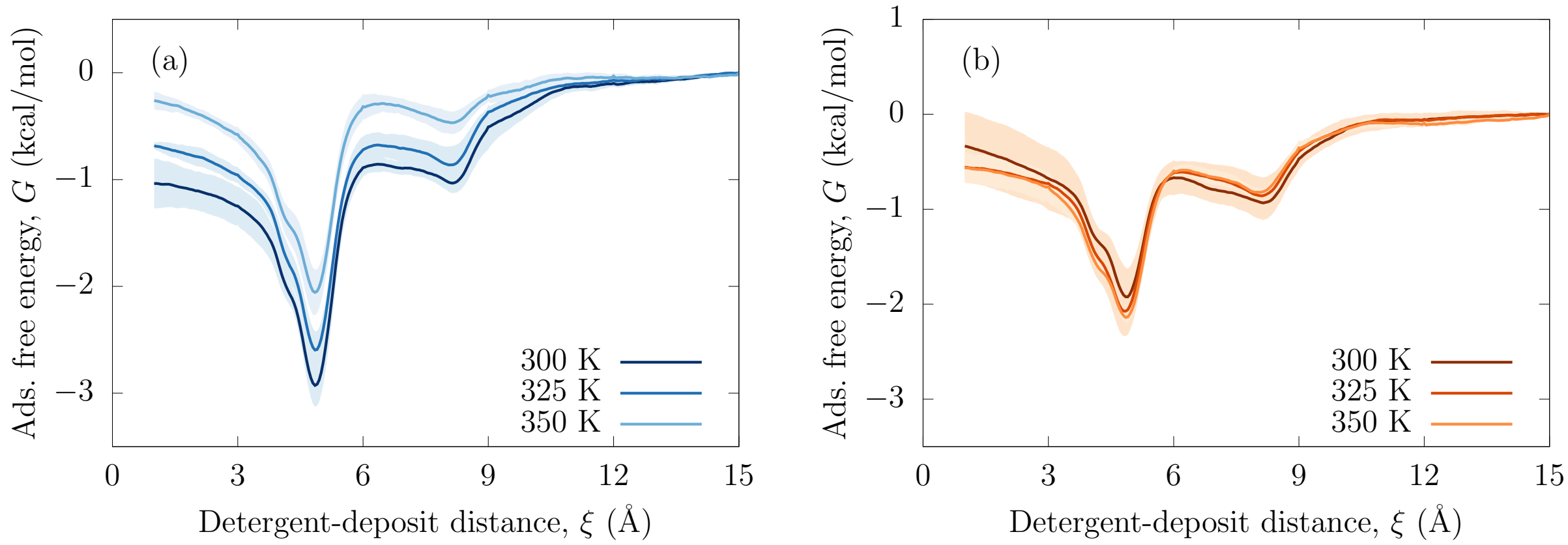

Figure S11: Adsorption PMF of the Mannich base DCA on circumovalene at 300, 325 and 350 K for (a) E15 and (b) E85 ethanol concentrations. Each profile is the mean of three independent ABF-MD runs and the shaded band denotes ±1 standard deviation across runs. At E15 the well deepens as the temperature is lowered, whereas at E85 the three profiles coincide with the run-to-run scatter.

Table S1: Adsorption free energy ($\mathrm{kcal\,mol^{-1}}$) at the PMF minimum ($\xi = 4.85$ Å) at three temperatures and its decomposition at 325 K, for E15, E85 and their difference, with its mean ± standard error over three independent runs (run-to-run SD = $\sqrt{3}$ SE). t and two-sided p on 7 degrees of freedom per concentration, 14 for the E85-E15 difference.

	E15	E85	E85 − E15
ΔG_{ads}, 300 K	-2.93 ± 0.12	-1.92 ± 0.18	$+1.00 \pm 0.21$
ΔG_{ads}, 325 K	-2.60 ± 0.10	-2.07 ± 0.15	$+0.53 \pm 0.18$
ΔG_{ads}, 350 K	-2.06 ± 0.12	-2.14 ± 0.03	-0.08 ± 0.13
$-T\Delta S_{\mathrm{ads}}$, 325 K	$+5.7 \pm 1.0$	-1.4 ± 1.2	-7.1 ± 1.5
	($t = 5.6$, $p = 0.0008$)	($t = -1.2$, $p = 0.26$)	($t = -4.6$, $p = 4 \times 10^{-4}$)
ΔH_{ads}, 325 K	-8.3 ± 1.0	-0.7 ± 1.2	$+7.6 \pm 1.5$

and the central value of $-T\Delta S$ changes sign along the profile, from about -1.8 $\mathrm{kcal\,mol^{-1}}$ in the contact well to $+1.0$ $\mathrm{kcal\,mol^{-1}}$ near 8.5 Å, both within one standard error of zero. The difference between the two concentrations is significant from contact to $\xi \approx 8.7$ Å. At E15 the threshold is thus exceeded by a wide margin over a contiguous range of 10 Å, and at E85 no bin reaches it even before allowing for the number of bins examined, so neither conclusion depends on having selected a single ξ.

The enthalpic regime found at E15 is a consequence of the chemical nature of the PAH deposit, and the small entropic term found at E85 need not hold for other surfaces. For the same Mannich base DCA adsorbing on hematite from an E15 solvent, ethanol coordinates

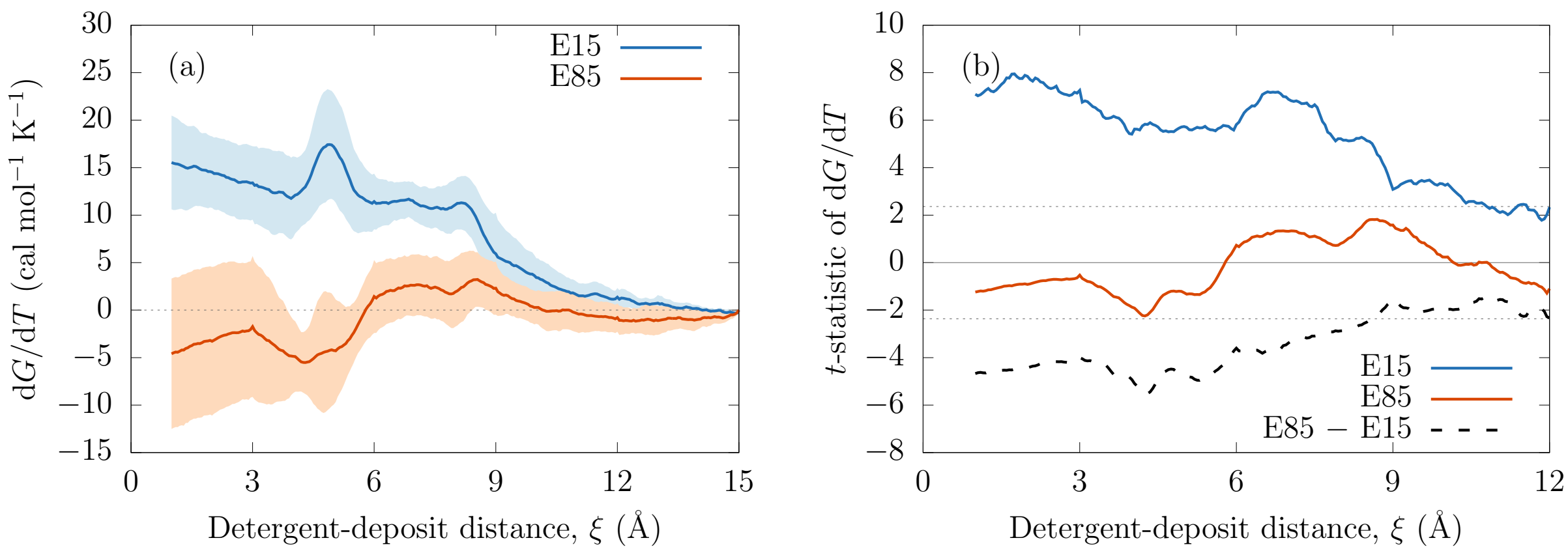

Figure S12: Entropy profile of the adsorption of the Mannich base DCA along the collective variable. (a) $\mathrm{d}G/\mathrm{d}T(\xi)$, the slope of the least-squares fit of $G(\xi, T)$ over 300, 325 and 350 K through all nine independent runs for E15 (blue) and E85 (orange); bands are ± 1 standard deviation propagated from the run-to-run scatter. b) t-statistic of the slope for E15 and E85, and of their difference (dashed), shown up to 12 Å beyond which the profiles are pinned to the bulk reference; dotted lines mark the two-sided 5 % thresholds.

directly to the surface iron sites and forms strongly ordered layers that the surfactant must displace upon adsorption.[7] Because a single adsorbing surfactant thereby liberates several ordered, surface-bound ethanol molecules into the disordered bulk, that process carries a substantial positive entropy and adsorption strengthens with temperature, consistent with the thermal disordering of the surface ethanol layer observed in the hematite study. On the hydrophobic PAH deposit considered here no such ordered surface layer forms: the ethanol hydrogen-bond network remains uniform up to the deposit (Fig. 10 of the main text) and ethanol structures around the non-polar groups of the solute rather than on the surface, so the entropy liberated on adsorption is correspondingly smaller. The two systems illustrate the same underlying physics: the entropic contribution to adsorption is set by how much solvent ordering is destroyed on binding. At the same time, it shows that the polarity of the surface decides whether that contribution merely offsets the entropic penalty of binding, as found here at E85, or drives adsorption outright, as on hematite.

References

(1) Comer, J.; Gumbart, J. C.; Hénin, J.; Lelievre, T.; Pohorille, A.; Chipot, C. The adaptive biasing force method: Everything you always wanted to know but were afraid to ask. *Journal of Physical Chemistry B* **2015**, *119*, 1129–1151.

(2) Fiorin, G.; Klein, M. L.; Hénin, J. Using collective variables to drive molecular dynamics simulations. *Molecular Physics* **2013**, *111*, 3345–3362.

(3) Day, T. J. F.; Patey, G. N. Ion solvation dynamics in binary mixtures. *The Journal of Chemical Physics* **1997**, *106*, 2782–2791.

(4) Fileti, E. E.; Chaudhuri, P.; Canuto, S. Relative strength of hydrogen bond interaction in alcohol-water complexes. *Chemical Physics Letters* **2004**, *400*, 494–499.

(5) Smith, D. E.; Haymet, A. D. J. Free energy, entropy, and internal energy of hydrophobic interactions: Computer simulations. *The Journal of Chemical Physics* **1993**, *98*, 6445–6454.

(6) Raschke, T. M.; Levitt, M. Nonpolar solutes enhance water structure within hydration shells while reducing interactions between them. *Proceedings of the National Academy of Sciences* **2005**, *102*, 6777–6782.

(7) Chia, C. L.; Alloway, R. M.; Jephson, I.; Clarke, S. M.; Filip, S. V.; Siperstein, F. R.; Avendaño, C. Competitive Adsorption of a Multifunctional Amine and Phenol Surfactant with Ethanol on Hematite from Nonaqueous Solution. *Journal of Physical Chemistry B* **2019**, *123*, 1375–1383.
